# Generalized-active-space pair-density functional theory: an efficient method to study large, strongly correlated, conjugated systems†
†Electronic supplementary information (ESI) available: Singlet–triplet energy gaps for CASSCF(4,4), CASSCF(8,8), DFP-1, DFP-3, and KS-DFT methods, numbers of CSFs with more significant figures, occupation numbers for HONO–1, HONO, LUNO and LUNO+1 for FP-1 and DFP-1, singlet–triplet energy gap (kcal mol^–1^) of decacene for DFP-1 partition with other geometries, singlet–triplet energy gap (kcal mol^–1^) of hexacene for different on-top functionals, molecular geometries, and absolute energies. See DOI: 10.1039/c6sc05036k



**DOI:** 10.1039/c6sc05036k

**Published:** 2017-01-19

**Authors:** Soumen Ghosh, Christopher J. Cramer, Donald G. Truhlar, Laura Gagliardi

**Affiliations:** a Department of Chemistry , Chemical Theory Center , Supercomputing Institute , University of Minnesota , 207 Pleasant Street SE , Minneapolis , MN 55455-0431 , USA . Email: gagliard@umn.edu ; Email: cramer@umn.edu ; Email: truhlar@umn.edu

## Abstract

Predicting ground- and excited-state properties of open-shell organic molecules by electronic structure theory can be challenging because an accurate treatment has to correctly describe both static and dynamic electron correlation. Strongly correlated systems, *i.e.*, systems with near-degeneracy correlation effects, are particularly troublesome. Multiconfigurational wave function methods based on an active space are adequate in principle, but it is impractical to capture most of the dynamic correlation in these methods for systems characterized by many active electrons. We recently developed a new method called multiconfiguration pair-density functional theory (MC-PDFT), that combines the advantages of wave function theory and density functional theory to provide a more practical treatment of strongly correlated systems. Here we present calculations of the singlet–triplet gaps in oligoacenes ranging from naphthalene to dodecacene. Calculations were performed for unprecedently large orbitally optimized active spaces of 50 electrons in 50 orbitals, and we test a range of active spaces and active space partitions, including four kinds of frontier orbital partitions. We show that MC-PDFT can predict the singlet–triplet splittings for oligoacenes consistent with the best available and much more expensive methods, and indeed MC-PDFT may constitute the benchmark against which those other models should be compared, given the absence of experimental data.

## Abstract 2

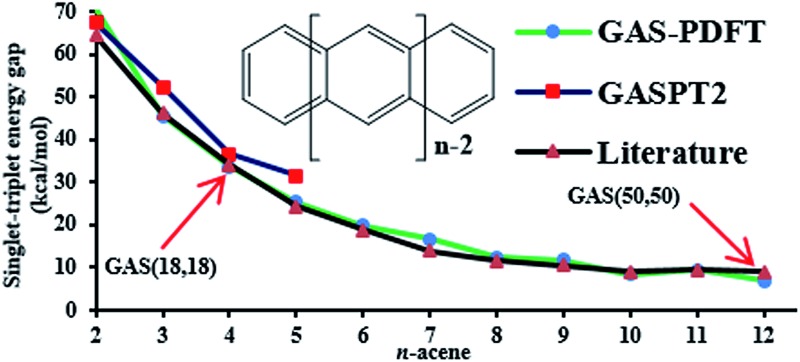
Predicting ground- and excited-state properties of open-shell organic molecules by electronic structure theory can be challenging because an accurate treatment has to correctly describe both static and dynamic electron correlation.

## 1 Introduction

Open-shell organic systems are important in several areas of chemical science including magnetism,^1–3^ photovoltaic materials,^4–6^ photochemistry,^7,8^ light-powered devices such as switching and sensing devices,^2,9^ and biological,^10,11^ biomimetic,^12,13^ and organometallic^14,15^ catalysis. However, studying their electronic structure is challenging because they often exhibit strong electron-correlation, *i.e.*, their electronic structure is strongly multiconfigurational due to near-degeneracy correlation, often called static correlation^16^ (systems of this type will be called multireference systems, and those without strong static correlation will be called single-reference systems). Quantitative treatments of open-shell systems require one to describe not only static correlation but also dynamic correlation, which arises from electrons avoiding one another at short range to minimize repulsion or correlating their motion at large distances leading to dispersion and dispersion-like forces.

**Fig. 1:**
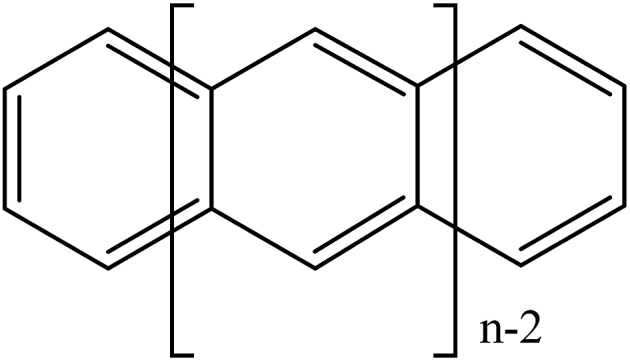
*n*-Acene series.

Organic molecules with open-shell singlet character are prime examples of strongly correlated systems. Oligoacenes (compounds consisting of several linearly fused benzene rings; see Fig. 1) have singlet ground states that develop increasing open-shell character with increasing length and are considered as prime test cases to study the performance of electronic structure methods for open-shell systems.^17–30^ These open-shell singlets may be considered to be diradicals for oligoacenes of moderate length, and polyradicals for still longer ones, with about two unpaired (or significantly partially unpaired) spins (one α, one β) for each five or so rings.^17,27^ In the last few decades, oligoacenes and their derivatives have also become of great interest for applications due to their charge transport properties,^31–34^ complex excited state dynamics,^35–38^ and electronic structure.^39^

A potentially transformative process for improving the efficiency of photovoltaic devices is singlet fission, in which a singlet exciton decays into two triplet excitons.^35–37^ Long oligoacenes are especially promising candidates for singlet fission due to their favourable crystal packing and electronic structure.^40,41^ The efficiency of singlet fission is strongly correlated with polyradical or diradical character of the ground state^42,43^ and with the singlet–triplet (ST) gap of the molecule.^44^ While short oligoacenes mostly have closed-shell ground states, longer acenes (beyond pentacene) develop open-shell character^17^ and the ground state can in principle be either a singlet or a triplet, with both having multireference character. Predicting the ground spin state and the singlet–triplet energy splitting is essential to understanding the singlet fission mechanism and to designing new materials.

An excellent overview of the electronic structure of oligoacenes and a review of the various theoretical treatments up to 2010 has been provided by Bettinger.^45^ Angliker *et al.* extrapolated available experimental UV-vis data and predicted all oligoacenes beyond nonacene to have a triplet ground state.^39^ This was supported by initial Kohn–Sham density functional theory (KS-DFT) calculations that considered only closed-shell singlet states,^46^ but when open-shell singlet states were considered employing a broken-symmetry formalism, KS-DFT calculations predicted singlet diradical states as ground states for oligoacenes beyond pentacene.^47^ Several electronic structure calculations including density matrix renormalization group (DMRG) calculations with a Pariser–Parr–Pople (PPP) Hamiltonian^48^ and with an *ab initio* Hamiltonian,^17^ two electron reduced density matrix (vRDM) calculations,^19^ and coupled cluster calculations^20,21^ also predicted all acenes up to dodecacene to have a singlet ground state, *i.e.*, the diradicals and polyradicals are antiferromagnetically coupled. More recently, Ibeji and Ghosh employed spin-flip methods to compute the singlet–triplet gaps of oligoacenes and through extrapolation of their data, they have shown that there is no singlet–triplet crossover for infinite chain acenes.^26^

Diradicals and polyradicals are strongly correlated, and one of the reasons behind these differences in the prediction of the spin ground state of long oligoacenes is that there have been no electronic structure methods that can reliably treat systems that are both strongly correlated and large. Two candidate methods are KS-DFT and coupled cluster theory. The KS-DFT method is affordable for large systems, and it represents open-shell singlets using broken-symmetry Slater determinants with electron spin component *M*_S_ equal to 0 and having two (or more) unpaired electrons with opposite spins in different molecular orbitals. These broken-symmetry states are a mixture of a singlet and a triplet state; if one were able to use an exact exchange–correlation functional, KS-DFT could give the exact results for the singlet using a broken-symmetry solution, but available approximate functionals tend to give an energy between that of the singlet and triplet. Although several methods have been advanced to extract the singlet state energy using the broken-symmetry solution,^49–52^ and although they are sometimes successful, they are not reliable. Coupled cluster theory can also in principle yield the correct singlet energy, but conventional coupled cluster theory uses a single-configuration reference wave function, and to make a treatment with such a reference state reliable for multireference systems often requires including triple and higher excitations, which is impractical for medium-sized and larger systems. Another way to treat biradicals and polyradicals with lower excitation levels is to use spin-flip (SF) approaches^26,53,54^ that take single-determinantal high spin states as the reference state.

An alternative with greater prospects for success in treating multireference systems is a method that adds dynamical correlation to a multiconfiguration self-consistent field (MCSCF) wave function^55^ that has the correct spin symmetry and is variationally optimized for both the orbitals used in the configuration state functions (CSFs) and the coefficients of the CSFs in a configuration interaction expansion of the wave function. There are several possible ways to choose the configurations in the CI expansion to try to balance accuracy (which can demand a large number of configurations) and affordability (which can demand a smaller number). Many of these are best explained by first considering the complete active space self-consistent field (CASSCF) method,^56^ which is a special case of MCSCF in which a full configuration interaction expansion (*i.e.*, one including all possible ways to assign the electrons to orbitals) of the wave function is constructed over a specified active space of *n* electrons and *m* orbitals with all the other orbitals either doubly occupied or vacant. However, the number of CSFs in this full-CI-within-a-window wave function increases exponentially with the active-space size and this approach already reaches its practical limit for closed-shell singlets when *n* = 18 and *m* = 18 and for open-shell states with similar *n* and *m* (depending on the state). This means that CASSCF calculations including all oligoacene valence-π orbitals in the active space are possible for at most up to tetracene. Therefore methods like the generalized active space SCF (GASSCF),^57,58^ the restricted active space SCF (RASSCF),^59–62^ the occupation restricted multiple active space (ORMAS),^63^ and split-GAS^64^ have been developed to remove many of the less important CSFs from the full CI. For example, in the GASSCF model, the active orbitals are placed into distinct subspaces, and accumulated minimum and maximum electron occupation numbers are applied to each subspace. Within a subspace, one includes all possible spin- and symmetry-adapted CSFs that can be constructed with this occupation number constraint plus, optionally, a restricted set of intersubspace excitations. This allows larger active spaces (larger *n* and *m*) than one can treat with CASSCF, while keeping the numbers of CFSs affordable.

Since an MCSCF calculation includes only a small fraction of the dynamic correlation, one must include the remaining dynamic correlation, which is necessary for chemical accuracy, by a post-SCF step. Until recently, the most affordable method that uses an MCSCF wave function as a reference for adding dynamic correlation in a post-SCF step has been complete active space second order perturbation theory (CASPT2)^65,66^ or restricted active space second order perturbation theory (RASPT2).^61^ Recently the capability has been developed to also carry out GASPT2 (ref. 67) calculations, although with the approximation that excitations into the orthogonal complement of the GAS space in the CAS space are omitted from the perturbed wave function. For this reason, the GASPT2 results presented in the following have only a subset of the second-order terms and hence are incomplete. However, it is still useful to report the GASPT2 results, because they are the only affordable calculations that one can perform at present using a perturbation treatment on top of a GASSCF wave function. Although these PT2 methods are capable of providing high accuracy,^68^ they are not suitable for large systems due to the rapid increase of computational cost and memory requirements.

As an alternative, we have recently developed a method called multiconfiguration pair-density functional theory (MC-PDFT).^69^ It is a new kind of density functional theory in which the electronic energy is calculated from the kinetic energy, density, and on-top pair density of a multiconfigurational wave function. The MC-PDFT method requires a reference calculation to generate an on-top pair density, which is the probability of finding two electrons at a given point in space. Whereas the density functional in KS-DFT depends on the spin-up electron density and the spin-down electron density and is called the exchange–correlation functional, the density functional in MC-PDFT depends on the total electron density and on-top pair density and is called the on-top density functional. MC-PDFT has been successfully used to compute excitation energies,^70–72^ barrier heights,^73^ and transition metal energetics.^74^ In these prior calculations, we have employed several different on-top density functionals that have usually given similar results. In the present article, we use only one of these, namely tPBE, which was defined in our first MC-PDFT paper.^69^

In general, any method that generates a two-body density matrix can be used as an MC-PDFT reference, although so far applications have been limited to using CASSCF and GASSCF wave functions, for which one may view MC-PDFT as a post-MCSCF method. For calculations based on GASSCF, a special case of GASSCF called the separated pair (SP) approximation has been defined and proven to be successful.^75^ SP is the special case in which no more than two orbitals are included in any GAS subspace and in which inter-subspace excitations are excluded. The SP approximation typically leads to far fewer configurations than CASSCF for the same *n* and *m*, and it has been successful for ground-state calculations. For excitation energies in the present paper, we explore alternative GASSCF partitions based on frontier orbitals.

MC-PDFT using CASSCF or GASSCF reference wave functions has been tested only for small systems. In the present study, we employed MC-PDFT to compute the singlet–triplet energy difference for oligoacenes ranging in length from naphthalene to dodecacene. A reasonable choice of active space for a π-conjugated system is an unabridged valence-π active space, with each occupied orbital having a correlating orbital, but a CASSCF with this active space reaches its limit at tetracene (18 electrons in 18 orbitals). On the other hand, within the GASSCF formalism, active spaces as large as 50 electrons in 50 orbitals can be employed. We here test the performance of MC-PDFT for various GASSCF wave functions with the aim of providing a general prescription on how to perform MC-PDFT calculations on large π-conjugated systems.

## 2 Computational methods

The geometries of the oligoacenes for both the singlet and triplet states were optimized using the B3LYP^76^ exchange–correlation functional and the 6-31G(d,p) basis set. For comparison with the present MC-PDFT calculations, we also carried out KS-DFT calculations with the 6-31+G(d,p) basis set with the PBE0 (ref. 77) exchange–correlation functional as well as with the PBE,^78^ one on which both PBE0 and tPBE are based. All KS-DFT calculations were performed with the unrestricted broken-symmetry approach (labeled “variational” in previous work^52^) using Gaussian 09.^79^

All CASPT2 and GASPT2 calculations were performed using the standard empirical IPEA^80^ shift value of 6.80 eV (0.25 a.u.) and an imaginary shift^81^ of 5.44 eV (0.2 a.u.). All MC-PDFT calculations were performed with the tPBE^69^ on-top functional. We have also performed ftPBE,^74^ tBLYP^69^ and ftBLYP^74^ calculations for hexacene to see the functional dependence of the MC-PDFT results. Results with these other functionals are reported in the ESI.† The 6-31+G(d,p) basis set was used for the CASSCF, GASSCF, CASPT2, GASPT2, and tPBE calculations. For all these calculations, Cholesky decomposition *via* resolution of the identity,^82^ was used to facilitate the computation of the two-electron integrals. All CASSCF, GASSCF, CASPT2, GASPT2, and tPBE calculations were performed in a locally modified version of Molcas 8.1.^83^

Oligoacenes ranging from naphthalene (*n*-acene with *n* = 2) to dodecacene (*n*-acene with *n* = 12) were investigated. All calculations were performed by imposing *D*_2h_ symmetry. In this point group, all singlet states considered belong to the ^1^A_g_ irreducible representation, and the triplet states belong to the ^3^B_3u_ irreducible representation. Adiabatic singlet–triplet energy gaps were determined as electronic energy differences between the energies calculated at the respective optimized geometries of the singlet ground state and the triplet state, whereas the vertical singlet–triplet gaps are calculated at the optimized singlet geometries. To calculate singlet–triplet gaps for infinite chain acenes for tPBE, we have fitted our data to an exponential decay of the form *a* + *b* exp(–*cx*) as done in ref. 26.

Several CASSCF and GASSCF active space choices were tested, as discussed next.

### CASSCF active space choices

For the CASSCF calculations, we use the notation CAS(*n*,*m*) where *n* is the number of active electrons, and *m* is the number of orbitals in the active space. In the paper, we report the performance of MC-PDFT and CASPT2 for a minimal active space, *i.e.*, CAS(2,2). Larger active spaces, in particular CAS(4,4) and CAS(8,8) were also considered; all results obtained for the larger complete active spaces are reported in the ESI.†

### GASSCF active spaces

**Fig. 2:**
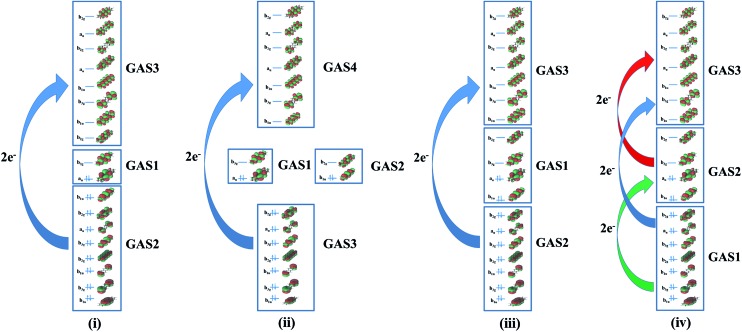
Pictorial representations of different generalized active space partitions for tetracene: (i) FP-1, (ii) DFP-1, (iii) WFP-1, and (iv) WFP-3.

All GASSCF active spaces include all valence π orbitals in the active space, but four GASSCF partitions are examined in which different choices are made for the accumulated minimum and maximum electron occupation numbers (Fig. 2).

#### Frontier partition with one set of interspace excitations (FP-1)

In this partition, GAS1 includes the HOMO and LUMO orbitals, GAS2 includes all other occupied π orbitals, and GAS3 includes all unoccupied π* orbitals. Single and double excitations are allowed within GAS1. Moreover, single and double excitations are allowed from GAS2 to GAS3, and no excitations are allowed in or out of GAS1.

#### Double-frontier partition with one set of interspace excitations (DFP-1)

In this partition, GAS1 includes the HOMO and LUMO orbitals, GAS2 includes the HOMO–1 and LUMO+1 orbitals, GAS3 includes all the other occupied π orbitals, and GAS4 includes all the remaining unoccupied π* orbitals. In this case single and double excitations are allowed within GAS1 and within GAS2; no intersubspace excitations are allowed for GAS1 and GAS2; single and double excitations are allowed from GAS3 to GAS4.

#### Wide-frontier partition with one set of interspace excitations (WFP-1)

In this partition, GAS1 includes the HOMO, LUMO, HOMO–1, and LUMO+1 orbitals, GAS2 includes all the other occupied π orbitals and GAS3 include all the other unoccupied π* orbitals. Thus GAS1 has four orbitals, and GAS2 and GAS3 each have (*n* – 4)/2 orbitals, where *n* is the number of valence π orbitals (*n* equals the number of carbon atoms in the oligoacene). All excitations are allowed within GAS1; single and double excitations are allowed from GAS2 to GAS3 and no intersubspace excitations are allowed for GAS1.

#### Wide-frontier partition with three sets of interspace excitations (WFP-3)

In this partition, labelling of GAS1 and GAS2 are switched with respect to the WFP-1 case, and GAS3 remains the same, but now single and double excitations are allowed from GAS1 to GAS2, GAS1 to GAS3, and GAS2 to GAS3. The minimum and maximum number of electrons in GAS1 are *n* – 6 and *n* – 4 respectively; and the minimum and maximum numbers of electrons in GAS1 + GAS2 are *n* – 2 and *n* respectively (so the maximum excitations into GAS3 is 2). All excitations are allowed within GAS2.

## 3 Highest-level available literature estimates and asymptotic limit

Literature values for vertical and adiabatic singlet–triplet gaps in oligoacenes from previous electronic structure calculations are reported in Table 1. The CCSD(T) values are obtained starting from restricted Hartree–Fock reference wave functions.^21^ Such CCSD(T) calculations become less reliable as oligoacene size increases because of the increasingly strongly correlated character. The DMRG values^17^ are based on complete active space configuration interaction without orbital optimization and with small basis sets, and for hexacene and smaller acenes they include three π* orbitals for each π orbital (for octacene and larger acenes they use the same active space as used here, *i.e.*, one π* orbital for each π orbital, but with the very small STO-3G basis set, and these results are not used for comparison here). The vRDM results^19^ are based on an active space containing all valence π electrons (as in some of the present calculations – see below). But neither DMRG nor vRDM attempt to recover all the dynamic correlation energy, which makes the accuracy of these results questionable. The multireference Moller–Plesset (MRMP) results^84^ for anthracene and tetracene are based on active spaces with 12 electrons in 12 orbitals where six π and six π* orbitals are included. Even though MRMP is capable of recovering dynamic correlation energy, such calculations with balanced active spaces are not affordable for larger acenes. Multireference configuration interaction singles and doubles with correction for quadruple and higher excitations (MRCISD + Q)^85^ results are obtained using reference states obtained from complete active space calculations using an active space of 8 electrons in 8 orbitals. The +Q correction approximately corrects for the underestimate of dynamic correlation energy by MRCISD as it only includes single and double excitations. We take the results in the vertical gap column and the average adiabatic gap column as highest-level available literature estimates, and in the following we report average absolute deviations from these values as mean unsigned deviations (MUDs).

**Table 1:** Highest-level available literature estimates from computational studies for vertical and adiabatic singlet–triplet energy gaps (kcal mol^–1^) in oligoacenes

Molecule	Vertical gap^*a*^	Adiabatic gap	Average adiabatic gap
Naphthalene	76.0	65.8^*a*^,63.5^*b*^,61.0^*c*^,67.3^*d*^	64.4^*f*^
Anthracene	56.8	48.2^*a*^,44.8^*b*^,44.0^*c*^,48.0^*d*^,46.1^*e*^	46.2^*f*^
Tetracene	40.4	33.5^*a*^,31.9^*b*^,31.9^*c*^,38.3^*d*^,34.8^*e*^	34.1^*f*^
Pentacene	31.3	25.3^*a*^,23.3^*b*^,23.4^*c*^,25.1^*d*^	24.3^*f*^
Hexacene	22.8	17.7^*a*^,17.6^*b*^,17.5^*c*^,21.9^*d*^	18.7^*f*^
Heptacene	18.1	13.4^*a*^,13.9^*b*^,14.5^*d*^	13.9^*f*^
Octacene	13.4	9.2^*a*^,11.5^*b*^,13.8^*d*^	11.5^*f*^
Nonacene	10.7	10.1^*b*^,10.6^*d*^	10.4^*f*^
Decacene	8.1	9.0^*b*^	9.0
Undecacene	7.1	9.4^*b*^	9.4
Dodecacene	NA^*g*^	8.9^*b*^	8.9
Infinite chain^*h*^			5.1

^*a*^CCSD(T)/CBS from ref. 21, where “CBS” denotes extrapolation to a complete one-electron basis set.

^*b*^vRDM/CBS from ref. 19.

^*c*^DMRG/DZ from ref. 17.

^*d*^π-MR-CISD + Q/CAS(8,8)/6-31G from ref. 85.

^*e*^MRMP/cc-pVDZ from ref. 84.

^*f*^Average of the values in adiabatic gap column.

^*g*^Not available.

^*h*^From ref. 26.

In ref. 26, the authors fit the best estimates of the adiabatic singlet–triplet gaps to an exponential decay of the form *a* + *b* exp(–*cx*), from which the extrapolated singlet–triplet gaps for infinitely long polyacenes are 5.06 kcal mol^–1^ for the best estimates and 5.37 kcal mol^–1^ for their best spin-flip results. We will compare our long chain singlet–triplet gap values to the value of 5.06 kcal mol^–1^.

## 4 Results and discussion

We first discuss the CASPT2 and tPBE results using the CAS(2,2) active space (Table 2; for additional CAS(4,4) and CAS(8,8) results, see Tables S1 and S2,† respectively). Both CASPT2 and tPBE predict a singlet ground state for all oligoacenes. From nonacene to dodecacene, the vertical singlet–triplet gap is lower than the adiabatic one, which cannot occur physically but it must be recalled that these energy differences are all computed using fixed B3LYP geometries (to facilitate comparison with prior predictions), *i.e.*, these are not vertical excitation energies for each different theory's ground-state geometry. This inversion of the energy ordering is observed for other active spaces as well, and it would be interesting in future work to optimize all geometries at the CASSCF, CASPT2, and tPBE levels; at present, however, this is not technically feasible. In any case, the reported MUD is meaningful since all vertical excitation values in the table (both our new calculations and the literature values) are computed for the same geometries.

**Table 2:** Singlet–triplet energy gap (kcal mol^–1^) for CAS(2,2) active space^*a*^

Acene	CASSCF		CASPT2		tPBE (CAS-PDFT)		Literature values^*b*^	
	Vert.	Ad.	Vert.	Ad.	Vert.	Ad.	Vert.^*c*^	Ad.^*d*^
Naphthalene	86.4	72.2	77.0	68.2	74.5	66.4	76.0	64.4
Anthracene	61.2	50.2	60.1	51.9	59.6	51.8	56.8	46.2
Tetracene	46.5	37.5	40.8	34.4	37.2	31.8	40.4	34.1
Pentacene	37.7	29.6	34.2	28.5	31.3	26.4	31.3	24.3
Hexacene	25.3	19.4	23.6	19.9	19.7	16.8	22.8	18.7
Heptacene	18.5	15.3	18.5	17.4	14.2	13.6	18.1	13.9
Octacene	10.0	7.7	14.0	13.6	9.6	9.7	13.4	11.5
Nonacene	8.8	6.4	11.3	11.5	5.8	6.6	10.7	10.4
Decacene	4.7	2.0	10.2	10.4	5.4	6.1	8.1	9.0
Undecacene	4.8	1.3	8.4	8.8	2.0	3.3	7.1	9.4
Dodecacene	2.8	–1.6	8.7	9.1	3.5	4.6	NA^*e*^	8.9
MUD^*f*^	4.1	5.1	1.3	2.2	3.1	3.0		

^*a*^Geometries are optimized using B3LYP/6-31G(d,p) level of theory (with broken-symmetry solutions). CASSCF, CASPT2, and tPBE calculations are performed using the 6-31+G(d,p) basis set.

^*b*^Highest-level available literature estimates.

^*c*^CCSD(T)/cc-pV∞Z from ref. 21.

^*d*^Average adiabatic gaps from Table 1.

^*e*^Not available.

^*f*^Mean unsigned deviation from highest-level available literature estimates.

Table 2 shows that both CASPT2 and tPBE with the (2,2) CASSCF active space agree remarkably well with the literature values up to decacene; however, tPBE predicts increasingly small gaps for undecacene and dodecacene, which is not in agreement with the best available literature values. Extrapolation of the CASPT2 and tPBE results give limiting singlet–triplet gap of 7.1 kcal mol^–1^ and 1.3 kcal mol^–1^ respectively for an infinite acene compared to the value of 5.1 kcal mol^–1^ noted above as a previous best estimate. We note that prior studies have indicated that it is important to add other π orbitals into the active space,^22^ especially for longer oligoacenes, to fully account for diradical and ultimately polyradical character. A systematic way to do this is to include all occupied and unoccupied valence π orbitals in the active space. However, such a large active space is not affordable beyond tetracene in the CASSCF formalism; instead, we do this in the present work with the GASSCF frontier partitions defined above. Table 3 reports the number of CSFs for the GASSCF and corresponding CASSCF wave functions, including all valence π orbitals; this table shows a tremendous reduction in CSFs for the GASSCF cases. We also performed GASPT2 calculations when computationally practical. Tables 4 and 5 show complete results for the smallest and largest CSF lists among the four GASSCF partitions, namely FP-1 and WFP-3. Complete results for the other CASSCF active spaces, for the other two partitions of the valence π GASSCF spaces, and for the KS-DFT calculations are given in the electronic (ESI†), and mean unsigned deviations for the CASSCF(2,2) active space, for all four frontier partitions of the valence π GASSCF spaces and for KS-DFT will be compared below.

**Table 3:** Number of CSFs for valence-π CASSCF and GASSCF calculations

Acene	CASSCF		FP-1		DFP-1		WFP-1		WFP-3	
	Singlet	Triplet	Singlet	Triplet	Singlet	Triplet	Singlet	Triplet	Singlet	Triplet
Naphthalene	4956	7440	182	235	256	369	500	735	866	1247
Anthracene	6.9 × 10^5^	1.3 × 10^6^	778	1134	1692	2745	3424	5555	4944	7843
Tetracene	∼10^8^		2382	3615	6296	1.1 × 10^4^	1.3 × 10^4^	2.2 × 10^4^	1.7 × 10^4^	2.8 × 10^4^
Pentacene	∼10^10^		5706	8898	1.7 × 10^4^	2.9 × 10^4^	3.5 × 10^4^	6.0 × 10^4^	4.3 × 10^4^	7.3 × 10^4^
Hexacene	∼10^13^		1.2 × 10^4^	1.9 × 10^4^	3.8 × 10^4^	6.6 × 10^4^	7.8 × 10^4^	1.3 × 10^5^	9.3 × 10^4^	1.6 × 10^5^
Heptacene	∼10^15^		2.2 × 10^4^	3.5 × 10^4^	7.4 × 10^4^	1.3 × 10^5^	1.5 × 10^5^	2.6 × 10^5^	1.8 × 10^5^	3.0 × 10^5^
Octacene	∼10^17^		3.7 × 10^4^	5.9 × 10^4^	1.3 × 10^5^	2.3 × 10^5^	2.7 × 10^5^	4.7 × 10^5^	3.1 × 10^5^	5.3 × 10^5^
Nonacene	∼10^20^		5.9 × 10^4^	9.5 × 10^4^	2.2 × 10^5^	3.8 × 10^5^	4.4 × 10^5^	7.8 × 10^5^	5.0 × 10^5^	8.6 × 10^5^
Decacene	∼10^22^		9.0 × 10^4^	1.5 × 10^5^	3.4 × 10^5^	6.0 × 10^5^	6.9 × 10^5^	1.2 × 10^6^	7.7 × 10^5^	1.3 × 10^6^
Undecacene	∼10^24^		1.3 × 10^5^	2.1 × 10^5^	5.0 × 10^5^	9.0 × 10^5^	1.0 × 10^6^	1.8 × 10^6^	1.1 × 10^6^	2.0 × 10^6^
Dodecacene	∼10^27^		1.9 × 10^5^	3.0 × 10^5^	7.3 × 10^5^	1.3 × 10^6^	1.5 × 10^6^	2.6 × 10^6^	1.6 × 10^6^	2.8 × 10^6^

**Table 4:** Singlet–triplet energy gaps (kcal mol^–1^) for FP-1 partitions^*a*^

Acene	(*n*,*m*)	GASSCF		GASPT2		tPBE (GAS-PDFT)		Literature values^*b*^	
		Vert.	Ad.	Vert.	Ad.	Vert.	Ad.	Vert.^*c*^	Ad.^*d*^
Naphthalene	(10,10)	85.0	68.5	80.8	67.3	77.6	70.6	76.0	64.4
Anthracene	(14,14)	66.5	55.2	60.2	52.2	51.3	45.5	56.8	46.2
Tetracene	(18,18)	46.2	36.1	44.2	36.5	39.0	33.6	40.4	34.1
Pentacene	(22,22)	43.1	33.8	38.3	31.4	29.7	25.3	31.3	24.3
Hexacene	(26,26)	27.6	20.5			22.9	19.7	22.8	18.7
Heptacene	(30,30)	22.2	18.3			17.3	16.5	18.1	13.9
Octacene	(34,34)	11.8	9.1			12.4	12.4	13.4	11.5
Nonacene	(38,38)	10.9	8.3			11.4	11.8	10.7	10.4
Decacene	(42,42)	6.7	3.7			7.7	8.3	8.1	9.0
Undecacene	(46,46)	6.5	2.7			8.7	9.4	7.1	9.4
Dodecacene	(50,50)	4.9	0.18			5.9	6.8	NA^*f*^	8.9
MUD^*e*^		4.9	5.1			1.5	1.6		

^*a*^Geometries are optimized using B3LYP/6-31G(d,p) level of theory. GASSCF, GASPT2 and tPBE calculations are performed using 6-31+G(d,p) basis set.

^*b*^Highest-level available literature estimates.

^*c*^CCSD(T)/cc-pV∞Z from ref. 21.

^*d*^Average adiabatic gaps from Table 1.

^*e*^Mean unsigned deviation.

^*f*^Not available. For vertical excitations MUD is calculated for the values from naphthalene to undecacene only.

**Table 5:** Singlet–triplet energy gaps (kcal mol^–1^) for WFP-3 partitions^*a*^

Acene	(*n*,*m*)	GASSCF		GASPT2		tPBE (GAS-PDFT)		Literature values^*b*^	
		Vert.	Ad.	Vert.	Ad.	Vert.	Ad.	Vert.^*c*^	Ad.^*d*^
Naphthalene	(10,10)	72.8	63.4	74.5	65.7	74.9	64.7	76.0	64.4
Anthracene	(14,14)	57.9	49.0	54.1	46.6	50.4	43.1	56.8	46.2
Tetracene	(18,18)	45.5	37.8	40.4	33.9	34.7	28.8	40.4	34.1
Pentacene	(22,22)	34.0	27.1			25.0	20.5	31.3	24.3
Hexacene	(26,26)	25.6	20.4			17.3	15.0	22.8	18.7
Heptacene	(30,30)	16.9	14.5			10.6	10.0	18.1	13.9
Octacene	(34,34)	12.4	10.7			5.4	6.4	13.4	11.5
Nonacene	(38,38)	9.8	8.4			4.4	5.0	10.7	10.4
Decacene	(42,42)	8.2	6.5			3.7	5.1	8.1	9.0
Undecacene	(46,46)	7.8	5.6			1.6	3.1	7.1	9.4
MUD^*e*^		1.9	2.2			5.7	4.1		

^*a*^Geometries are optimized using B3LYP/6-31G(d,p) level of theory. CASSCF, GASPT2 and tPBE calculations are performed using 6-31+G(d,p) basis set.

^*b*^Highest-level available literature estimates.

^*c*^CCSD(T)/cc-pV∞Z from ref. 21.

^*d*^Average adiabatic gaps from Table 1.

^*e*^Mean unsigned deviation.

Table 4 shows that tPBE with the FP-1 partition agrees extremely well with literature values; in particular, the MUDs are respectively 1.5 kcal mol^–1^ and 1.6 kcal mol^–1^ for vertical and adiabatic singlet–triplet gaps. It is also encouraging to notice that tPBE predicts an asymptotic value of 7.6 kcal mol^–1^ compared to the reference value of 5.1 kcal mol^–1^. The incomplete GASPT2 calculations and the tPBE calculations predict very similar results for naphthalene, but they differ more for anthracene and tetracene.

Table 5 shows that for WFP-3 calculations, which include more configurations in the CI expansion because single and double excitations between GAS spaces are allowed, tPBE again agrees reasonably well with the literature values, with MUDs of 5.7 kcal mol^–1^ and 4.1 kcal mol^–1^ for vertical and adiabatic singlet–triplet gaps, respectively. These results are a little worse than with the smaller CSF list of FP-1 when compared to the literature values. WFP-3 predicts the asymptotic gap of 1.9 kcal mol^–1^ which is narrower than FP-1. Note that GASPT2 calculations could only be performed up to tetracene with the WFP-3 partition because of unaffordable resource demand (maximum available runtime of 240 hours) for the larger systems. Nevertheless, it is interesting that tPBE and GASPT2 agree quite well with each other for the first three systems. Note that GASSCF calculations with this larger CI expansion are affordable only up to undecacene as such calculations for dodecacene would require longer time than maximum available runtime of 240 hours.

With wave function methods, one would expect – all other things being equal – more accurate results with a larger CI expansion in the active space, but it is well known that this is not always the case because it is equally important or more important that the active space includes the qualitatively important near-degeneracy effects in a well-balanced way as that it is large. The same balance is important in MC-PDFT, and the results with the larger configuration lists are not always more accurate. With that caveat, we note that for both wide-frontier partitions (WFP-1 and WFP-3), tPBE systematically predicts smaller vertical and adiabatic singlet–triplet gaps for longer acenes with respect to the literature values. This is not surprising, though, since the DMRG and v2RDM literature values were all obtained without taking into account the total dynamic correlation. In our calculations, dynamic correlation is recovered using the on-top functional of pair-density functional theory. It has been predicted previously that the inclusion of dynamic correlation effects would decrease the singlet–triplet gaps by a few kcal mol^–1^.^17^ Thus, while the reported MUD for the WFP-3 partition is larger than that for the FP-1 partition, the predictions may be more accurate for the former and may indeed constitute the set of reference values against which other models should be compared. From a chemical standpoint, it is particularly significant that neither the tPBE calculations with the (2,2) CASSCF space nor the tPBE calculations with any of the frontier-orbital partitions of active spaces including all π orbitals show a singlet–triplet crossover up to dodecacene.

Table S8 in the ESI† shows results with other on-top functionals, and it is encouraging that Table S8† shows that the results also do not depend strongly on the choice of on-top functional.

Table 6 compares MC-PDFT mean unsigned deviations for CASSCF(2,2) reference wave functions and all four GASSCF partitions of the valence π active space. It is encouraging that the results do not depend strongly on the MCSCF wave function. It is interesting that the results are even slightly better on average for the frontier partitions involving smaller CSF lists, especially when we recall the caveats associated with the dependence of the results on the size of the configuration list and with the unknown accuracy of the literature values.

**Table 6:** Mean unsigned deviations (kcal mol^–1^) for singlet–triplet energy gaps

Multireference methods					
	CSFs	MCSCF		tPBE	
		Vert.	Ad.	Vert.	Ad.
CAS(2,2)	2(singlet), 1(triplet)	4.1	5.1	3.1	3.0
FP-1	182–3.0 × 10^5^	4.9	5.1	1.5	1.6
DFP-1	256–1.3 × 10^6^	5.3	3.5	3.4	2.9
WFP-1	500–2.6 × 10^6^	7.0	4.9	3.3	2.5
WFP-3	866–2.6 × 10^6^	1.9	2.2	5.7	4.1

Kohn–Sham density functional theory				
CSFs	PBE		PBE0	
	Vert.	Ad.	Vert.	Ad.
1(singlet), 1(triplet)	8.4	7.2	5.7	4.3

**Fig. 3:**
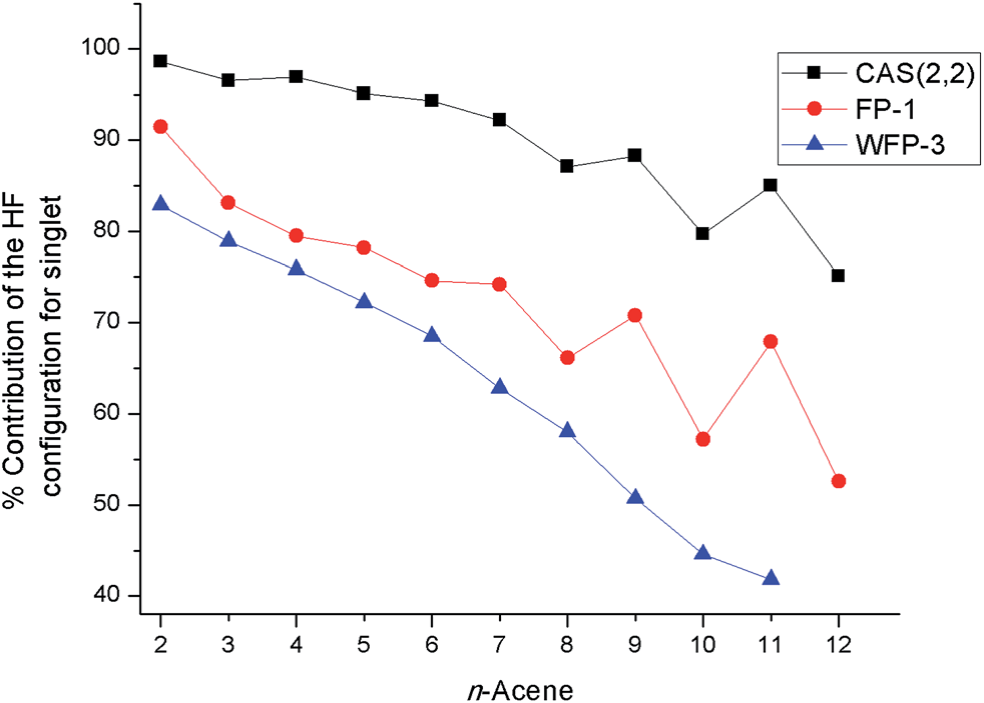
Percentage of the HF configuration for the singlet state for different acenes.

The percentage contribution of the most dominant configuration (the nominal HF configuration) for the singlet state decreases along the series (Fig. 3) suggesting the presence of significant multiconfigurational character for longer oligoacenes. The oscillations in Fig. 3 are reminiscent of that for the fundamental excitation gap of oligoacenes, that Korytár *et al.* ^86^ have attributed to a Dirac-like cone in the band structure. However, the smooth decrease in the HF configuration weight observed with the more complete WFP-3 suggests that the observed oscillations with fewer CSFs may alternatively be an artefact of a small active space.

**Fig. 4:**
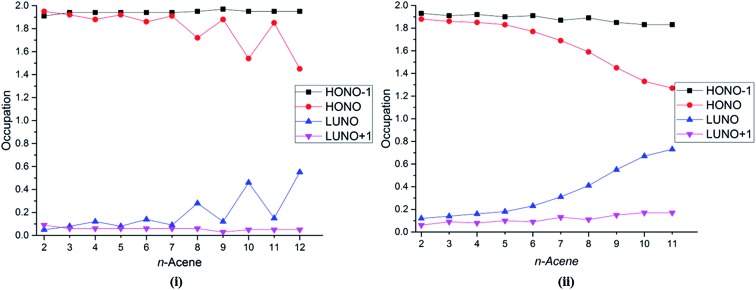
Occupation number of the HONO–1, HONO, LUNO, and LUNO+1 orbitals for different acenes for (i) FP-1, and (ii) WFP-3.

Several studies have discussed the diradical and polyradical character of long oligoacenes.^17,19,22,27^ This character can be analyzed in terms of the occupation numbers of the GASSCF natural orbitals (NOs). Fig. 4 shows the occupation number of the HONO–1, HONO, LUNO, and LUNO+1 for different oligoacenes for the FP-1 and WFP-3 partitions of the active space. Here – if *n* denotes the number of valence π electrons – HONO denotes the NO with occupation number *n*/2 when the orbitals are ordered by decreasing occupation number, and LUNO denotes orbital (*n*/2) + 1; HONO–1 denotes orbital (*n*/2) – 1, and LUNO+1 denotes orbital (*n*/2) + 2. The LUNO occupation number usually increases along the series, but again the trend is not monotonic. No symmetry switch occurs between the HONO and LUNO up to dodecacene, contrary to the observations in ref. 19 and 27. Interestingly for WFP-3, the occupation number of LUNO+1 also tends to increase, although at a slower rate than the LUNO occupation. An increasing occupation of the LUNO+1 is a signature of growing polyradical character.^17^ Again, significantly reduced oscillatory behaviour with WFP-3 is possibly attributable to the more complete nature of this active space.

**Fig. 5:**
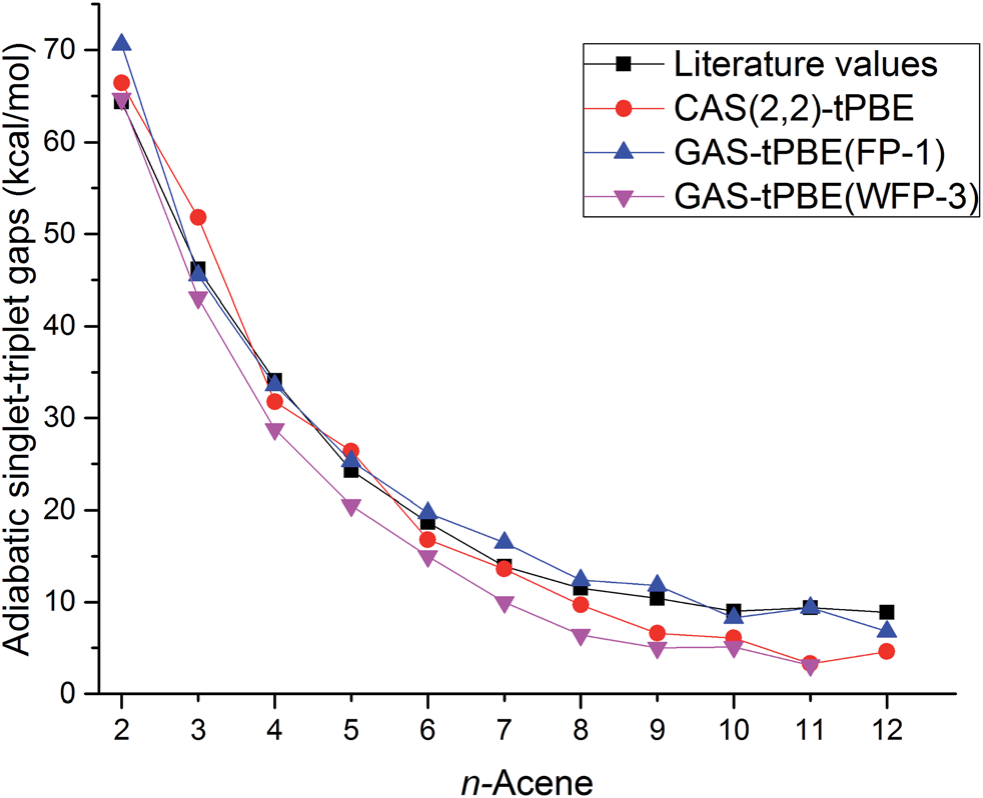
Adiabatic singlet–triplet gaps (kcal mol^–1^) for oligoacenes. Literature values are average adiabatic singlet–triplet gaps from Table 1.

The adiabatic singlet–triplet gaps obtained for tPBE using CAS(2,2), FP-1, and WFP-3 active spaces, and the literature values are compared in Fig. 5. With both CAS(2,2) and FP-1 reference wave functions, tPBE results match reasonably well with the literature values, while with WFP-3 reference wave functions, tPBE predicts systematically smaller adiabatic singlet–triplet gaps than the literature values, which may reflect better accounting for the differential effects of correlation on the two state energies.

## 5 Concluding remarks

In the present paper, we used the recently developed MC-PDFT method to compute the singlet–triplet gaps in oligoacenes ranging from naphthalene to dodecacene. We showed that the tPBE on-top density functional performs well with both minimal and valence-π active spaces. Using generalized active space reference wave functions, we were able to perform calculations with orbitally optimized active spaces up to 50 electrons and 50 orbitals and with dynamic correlation included by an on-top density functional. Even though it is not easy to generalize prescriptions for partitioning the active space, because the best partitions are expected to be system- and problem-dependent, the various frontier partitions used here may be useful for other similar problems as well as for oligoacenes. Our first conclusion is that the results are moderately sensitive to active space choice. Our second conclusion is that even small active spaces can yield good results and active spaces with sparse GAS partitioning can yield useful accuracy. However, it is not possible to draw further conclusions at this stage because, in the absence of experimental measurements, we have no way to assess the accuracy of the various high-level available literature values, and thus it is not possible to determine which active space provides the most accurate results. To amplify on that point, it may well be that one of the computational protocols employed herein is the most accurate of all methods to date, but a validation of that possible hypothesis will require measurement or demonstrably more complete calculations.

The GASSCF method has great potential to generate physically meaningful wave functions for large systems, and MC-PDFT based on these wave functions can provide chemical accuracy. Overall this study provides a framework in terms of both wave function theory and density functional theory that can be extended to study ground- and excited-state properties of organic electronic materials and other problems involving large, strongly correlated, conjugated systems.
