# Supplementary material for: Generalized-active-space pair-density functional theory: an efficient method to study large, strongly correlated, conjugated systems
Source: Chem Sci. 2017 Jan 19;8(4):2741–50. doi: 10.1039/c6sc05036k (PMC5433034; doi:10.1039/c6sc05036k)
Supplement: Supplementary file 1 [file SC-008-C6SC05036K-s001.pdf]

Electronic Supplementary Material (ESI) for Chemical Science.
This journal is © The Royal Society of Chemistry 2017

ELECTRONIC SUPPLEMENTARY INFORMATION

DECEMBER 25, 2016

Generalized-Active-Space Pair-Density Functional Theory: An Efficient Method to Study Large, Strongly Correlated, Conjugated Systems

Soumen Ghosh, Christopher J. Cramer*, Donald G. Truhlar*, Laura Gagliardi*

Department of Chemistry, Chemical Theory Center, and Supercomputing Institute, University of Minnesota, 207 Pleasant Street SE, Minneapolis, MN 55455-0431, USA.

E-mail: gagliardi@umn.edu; cramer@umn.edu; truhlar@umn.edu

Contents

Table S1. Singlet-triplet energy gaps (kcal/mol) for CAS(4,4) active space S-2

Table S2. Singlet-triplet energy gaps (kcal/mol) for CAS(8,8) active space S-3

Table S3. Number of CSFs for CASSCF and GASSCF calculations S-4

Table S4. Singlet-triplet energy gaps (kcal/mol) DFP-1 partitions S-5

Table S5. Singlet-triplet energy gaps (kcal/mol) for WFP-1 partitions S-6

Figure S1. Occupation numbers for HONO-1, HONO, LUNO and LUNO+1 for (i) FP-1 and (ii) DFP-1 S-7

Table S6. Singlet-triplet energy gaps (kcal/mol) for KS-DFT S-8

Table S7. Singlet-triplet energy gap (kcal/mol) of decacene for DFP-1 partition with other geometries S-9

Table S8. Singlet-triplet energy gap (kcal/mol) of hexacene using different on-top functionals S-9

Molecular geometries in Å S-10

Absolute energies in hartrees S-27

Table S1. Singlet-triplet energy gaps (kcal/mol) for CAS(4,4) active space

Acene	CASSCF		CASPT2		tPBE (CAS-PDFT)		Literature values[a]	
	Vertical	Adiabatic	Vertical	Adiabatic	Vertical	Adiabatic	Vertical[b]	Adiabatic[c]
Napthalene	93.7	80.4	75.5	67.9	70.7	62.6	76.0	64.4
Anthracene	69.7	59.5	60.3	51.9	55.9	47.8	56.8	46.2
Tetracene	52.5	45.8	41.3	34.8	33.9	28.1	40.4	34.1
Pentacene	32.8	25.5	30.6	25.5	25.5	21.2	31.3	24.3
Hexacene	29.3	23.4	26.2	22.3	22.2	18.9	22.8	18.7
Heptacene	11.3	8.7	16.7	15.4	11.9	10.9	18.1	13.9
Octacene	16.3	14.0	15.4	14.9	10.2	10.1	13.4	11.5
Nonacene	12.6	10.9	9.9	9.8	2.9	2.9	10.7	10.4
Decacene	11.9	9.3	10.5	10.6	4.6	5.2	8.1	9.0
Undecacene	9.6	6.9	7.5	7.6	0.8	1.3	7.1	9.4
Dodecacene	8.2	3.4	4.4	10.8	-5.3	4.9	NA[e]	8.9
MUD[d]	6.9	5.8	1.6	2.3	4.6	3.7		

Geometries are optimized using B3LYP/6-31G(d,p) level of theory. CASSCF, CASPT2 and tPBE calculations are performed using 6-31+G(d,p) basis set. [a] Highest-level available literature estimates. [b]CCSD(T)/cc-pV∞Z from ref 1. [c]Average adiabatic gaps from Table 1. [d]Mean unsigned deviation. [e]Not available. For vertical excitations MUD is calculated for the values from naphthalene to undecacene only.

Table S2. Singlet-triplet energy gaps (kcal/mol) for CAS(8,8) active space

Acene	CASSCF		CASPT2		tPBE (CAS-PDFT)		Literature values[a]	
	Vertical	Adiabatic	Vertical	Adiabatic	Vertical	Adiabatic	Vertical[b]	Adiabatic[c]
Napthalene	75.6	67.2	74.3	65.8	76.0	66.1	76.0	64.4
Anthracene	61.5	51.0	54.0	47.3	51.9	46.0	56.8	46.2
Tetracene	38.9	28.5	43.4	37.3	42.5	37.4	40.4	34.1
Pentacene	40.1	33.8	28.4	22.9	23.6	18.3	31.3	24.3
Hexacene	17.1	10.1	28.4	24.6	26.4	23.6	22.8	18.7
Heptacene	7.2	4.0	18.1	16.5	18.4	17.4	18.1	13.9
Octacene	13.0	11.4	11.2	10.5	7.6	7.3	13.4	11.5
Nonacene	11.7	9.7	9.8	9.5	6.8	6.8	10.7	10.4
Decacene	9.8	7.6	11.6	11.8	6.9	7.2	8.1	9.0
Undecacene	13.8	10.4	7.6	7.1	0.9	1.1	7.1	9.4
Dodecacene	11.0	6.7	3.4	10.7	-5.4	5.3	NA[e]	8.9
MUD[d]	4.2	4.2	2.3	2.2	3.6	3.7		

Geometries are optimized using B3LYP/6-31G(d,p) level of theory. CASSCF, CASPT2 and tPBE calculations are performed using 6-31+G(d,p) basis set. [a] Highest-level available literature estimates. [b]CCSD(T)/cc-pV∞Z from ref 1. [c]Average adiabatic gaps from Table 1. [d]Mean unsigned deviation. [e]Not available. For vertical excitations MUD is calculated for the values from naphthalene to undecacene only.

Table S3. Number of CSFs for CASSCF and GASSCF calculations with the valence π active space (this table is like Table 3 in the article proper, but it has the full number of digits.)

Molecule	CASSCF		FP-1		DFP-1		WFP-1		WFP-3	
	Singlet	Triplet	Singlet	Triplet	Singlet	Triplet	Singlet	Triplet	Singlet	Triplet
Napthalene	4956	7440	182	235	256	369	500	735	866	1247
Anthracene	691335	1252720	778	1134	1692	2745	3424	5555	4944	7843
Tetracene	$\sim 10^{8}$		2382	3615	6296	10641	12828	21563	16846	27783
Pentacene	$\sim 10^{10}$		5706	8898	17052	29373	34832	59527	43220	72703
Hexacene	$\sim 10^{13}$		11750	18563	37928	66085	77548	133943	92706	157955
Heptacene	$\sim 10^{15}$		21666	34590	73924	129737	151224	262963	176080	302559
Octacene	$\sim 10^{17}$		36878	59239	131040	231117	268148	468451	306158	529243
Nonacene	$\sim 10^{20}$		58978	95250	216284	382837	442672	775967	497820	864431
Decacene	$\sim 10^{22}$		89814	145563	337672	599333	691212	1214767	768010	1338243
Undecacene	$\sim 10^{24}$		131418	213678	504228	896865	1032248	1817803	1135736	1984495
Dodecacene	$\sim 10^{27}$		186062	303215	725984	1293517	1486324	2621723	1622070	2840699

Table S4. Singlet-triplet energy gaps (kcal/mol) for DFP-1 partitions

Acene	(*n,m*)	GASSCF		GASPT2		tPBE (GAS-PDFT)		Literature values[a]	
		Vertical	Adiabatic	Vertical	Adiabatic	Vertical	Adiabatic	Vertical[b]	Adiabatic[c]
Napthalene	(10,10)	70.7	62.1	75.0	66.0	76.9	62.6	76.0	64.4
Anthracene	(14,14)	69.1	57.8	59.9	51.9	48.1	42.1	56.8	46.2
Tetracene	(18,18)	42.5	32.8	41.6	36.5	36.8	32.9	40.4	34.1
Pentacene	(22,22)	37.7	29.6			29.9	24.9	31.3	24.3
Hexacene	(26,26)	22.5	15.6			28.4	24.8	22.8	18.7
Heptacene	(30,30)	21.1	17.6			14.0	13.0	18.1	13.9
Octacene	(34,34)	19.3	16.7			12.7	12.4	13.4	11.5
Nonacene	(38,38)	15.0	12.7			6.3	6.3	10.7	10.4
Decacene	(42,42)	15.0	12.2			6.6	6.9	8.1	9.0
Undecacene	(46,46)	13.2	9.9			3.6	3.7	7.1	9.4
Dodecacene	(50,50)	13.5	8.9			4.4	4.9	NA[e]	8.9
MUD[d]		5.3	3.5			3.4	2.9		

Geometries are optimized using B3LYP/6-31G(d,p). GASSCF, AGASPT2 and tPBE calculations are performed using the 6-31+G(d,p) basis set. [a]Highest-level available literature estimates. [b]CCSD(T)/cc-pV∞Z from ref 1. [c]Average adiabatic gaps from Table 1. [d]Mean unsigned deviation. [e]Not available. For vertical excitations MUD is calculated for the values from naphthalene to undecacene only.

Table S5. Singlet-triplet energy gaps (kcal/mol) for WFP-1 partitions

Molecule	(*n,m*)	GASSCF		GASPT2		tPBE (GAS-PDFT)		Literature values[a]	
		Vertical	Adiabatic	Vertical	Adiabatic	Vertical	Adiabatic	Vertical[b]	Adiabatic[c]
Napthalene	(10,10)	72.2	64.0	76.2	67.4	77.4	63.1	76.0	64.4
Anthracene	(14,14)	69.8	59.1	60.0	52.6	47.9	42.2	56.8	46.2
Tetracene	(18,18)	42.0	31.9	40.9	35.9	35.8	32.6	40.4	34.1
Pentacene	(22,22)	45.0	37.2			28.4	23.3	31.3	24.3
Hexacene	(26,26)	31.4	24.8			24.3	21.1	22.8	18.7
Heptacene	(30,30)	24.6	21.3			14.9	13.8	18.1	13.9
Octacene	(34,34)	20.5	18.0			13.1	13.1	13.4	11.5
Nonacene	(38,38)	14.6	12.5			6.3	6.5	10.7	10.4
Decacene	(42,42)	14.9	12.3			6.7	7.3	8.1	9.0
Undecacene	(46,46)	12.4	9.4			3.2	3.7	7.1	9.4
Dodecacene	(50,50)	13.0	8.6			4.0	4.9	NA[e]	8.9
MUD[d]		7.0	4.9			3.3	2.5		

Geometries are optimized using B3LYP/6-31G(d,p). GASSCF, AGASPT2 and tPBE calculations are performed using the 6-31+G(d,p) basis set. [a]Highest-level available literature estimates. [b]CCSD(T)/cc-pV∞Z from ref 1. [c]Average adiabatic gaps from Table 1. [d]Mean unsigned deviation. [e]Not available. For vertical excitations MUD is calculated for the values from naphthalene to undecacene only.

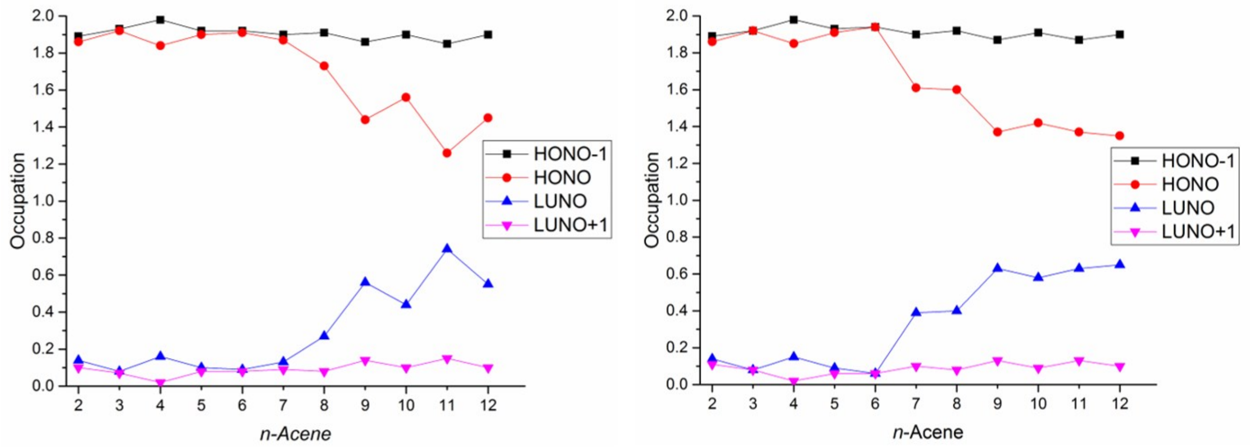

(i) **(ii)**

Figure S1. Occupation number for HONO-1, HONO, LUNO and LUNO+1 for (i) DFP-1 and (ii) WFP-1

Table S6. Singlet-triplet energy gaps (kcal/mol) for KS-DFT

Acene	PBE (KS-DFT)		PBE0 (KS-DFT)		B3LYP (KS-DFT)		Literature values[a]	
	Vertical	Adiabatic	Vertical	Adiabatic	Vertical	Adiabatic	Vertical[b]	Adiabatic[c]
Napthalene	78.2	60.4	71.7	62.1	71.9	62.1	76.0	64.4
Anthracene	46.7	40.0	48.6	41.1	49.0	41.5	56.8	46.2
Tetracene	31.8	26.5	33.1	26.9	33.6	27.6	40.4	34.1
Pentacene	21.6	17.2	22.3	16.9	22.9	17.9	31.3	24.3
Hexacene	13.4	10.6	14.4	10.7	14.3	10.9	22.8	18.7
Heptacene	6.3	5.6	9.4	8.0	8.4	7.2	18.1	13.9
Octacene	2.9	2.9	7.7	7.1	6.1	5.6	13.4	11.5
Nonacene	1.6	1.9	7.5	7.1	5.4	5.1	10.7	10.4
Decacene	1.3	1.7	8.2	7.8	5.5	5.3	8.1	9.0
Undecacene	1.6	2.1	9.5	9.0	6.2	6.0	7.1	9.4
Dodecacene	2.2	2.7	11.3	10.5	7.4	6.9	NA[e]	8.9
MUD[d]	8.4	7.2	5.7	4.3	6.1	5.0		

Geometries are optimized using B3LYP/6-31G(d,p). KS-DFT singlet-triplet energies are calculated using the 6-31+G(d,p) basis set. [a]Highest-level available literature estimates. [b]CCSD(T)/cc-pV∞Z from ref 1. [c]Average adiabatic gaps from Table 1. [d]Mean unsigned deviation. [e] Not available. For vertical excitations MUD is calculated for the values from naphthalene to undecacene only.

Table S7. Singlet-triplet energy gap (kcal/mol) of decacene for DFP-1 partition with other geometries

Decacene geomtery[a]	GASSCF		tPBE (MC-PDFT)		References	
	Vertical	Adiabatic	Vertical	Adiabatic	Vertical[b]	Adiabatic[c]
M06-L $\langle S^2 \rangle = 1.27$	15.0	13.6	6.5	6.7	8.1	9.0
M06 $\langle S^2 \rangle = 1.58$	15.0	11.3	6.5	7.1		

GASSCF and tPBE calculations are performed using the 6-31+G(d,p) basis set. [a]Decacene geometries are calculated using indicated exchange-correlation functionals and the 6-31G(d,p) basis set. $\langle S^2 \rangle$ values are for optimized singlet geometries. [b]CCSD(T)/cc-pV∞Z from ref 1. [c]v2RDM/cc-pV∞Z from ref 2.

Table S8. Singlet-triplet energy gap (kcal/mol) of hexacene using various on-top functionals.

Active space	tPBE		ftPBE		tBLYP		ftBLYP		Literature	
	Vertical	Adiabatic	Vertical	Adiabatic	Vertical	Adiabatic	Vertical	Adiabatic	Vertical[a]	Adiabatic[b]
CAS(2,2)	19.7	16.8	20.5	17.6	19.7	16.8	20.7	17.9	22.8	17.6
CAS(4,4)	22.2	18.9	22.4	19.1	22.2	18.9	22.6	19.4		
CAS(8,8)	26.4	23.6	24.4	21.3	26.4	23.6	25.1	22.1		
FP-1	22.9	19.7	22.6	19.4	22.9	19.8	22.9	19.8		
DFP-1	28.4	24.8	27.9	24.4	28.4	24.8	28.5	25.0		
WFP-1	24.3	21.1	24.2	20.9	24.3	21.1	24.5	21.3		
WFP-3	17.3	15.0	17.3	14.9	17.3	15.1	17.5	15.2		

Geometries are optimized using UB3LYP/6-31G(d,p) level of theory. CASSCF and tPBE calculations are performed using 6-31+G(d,p) basis set. [a]CCSD(T)/cc-pV∞Z from ref 1. [b]Average adiabatic gap from table 1.

Molecular geometries in Å

Singlet Geometries (B3LYP/6-31G**)

Napthalene

C	-1.244593000	1.402439000	0.000000000
C	-2.433051000	0.708272000	0.000000000
C	-2.433051000	-0.708272000	0.000000000
C	-1.244593000	-1.402439000	0.000000000
C	0.000000000	-0.716921000	0.000000000
C	0.000000000	0.716921000	0.000000000
H	-1.242226000	2.489450000	0.000000000
H	-3.377109000	1.245176000	0.000000000
H	-3.377109000	-1.245176000	0.000000000
H	-1.242226000	-2.489450000	0.000000000
C	1.244593000	1.402439000	0.000000000
C	2.433051000	0.708272000	0.000000000
H	3.377109000	1.245176000	0.000000000
C	2.433051000	-0.708272000	0.000000000
H	3.377109000	-1.245176000	0.000000000
C	1.244593000	-1.402439000	0.000000000
H	1.242226000	-2.489450000	0.000000000
H	1.242226000	2.489450000	0.000000000

Anthracene

C	0.000000000	1.403683000	0.000000000
C	-1.223603000	0.722675000	0.000000000
C	-1.223603000	-0.722675000	0.000000000
C	0.000000000	-1.403683000	0.000000000
C	1.223603000	-0.722675000	0.000000000
C	1.223603000	0.722675000	0.000000000
H	0.000000000	2.491422000	0.000000000
H	0.000000000	-2.491422000	0.000000000
C	2.478998000	1.406917000	0.000000000
C	3.659824000	0.712921000	0.000000000
H	4.605674000	1.246544000	0.000000000
C	3.659824000	-0.712921000	0.000000000
H	4.605674000	-1.246544000	0.000000000
C	2.478998000	-1.406917000	0.000000000
H	2.476980000	-2.493805000	0.000000000
H	2.476980000	2.493805000	0.000000000
C	-2.478998000	1.406917000	0.000000000
C	-3.659824000	0.712921000	0.000000000
H	-4.605674000	1.246544000	0.000000000
C	-3.659824000	-0.712921000	0.000000000
H	-4.605674000	-1.246544000	0.000000000
C	-2.478998000	-1.406917000	0.000000000
H	-2.476980000	-2.493805000	0.000000000
H	-2.476980000	2.493805000	0.000000000

Tetracene

C	-1.235102000	1.406546000	0.000000000
C	-2.450052000	0.725940000	0.000000000
C	-2.450052000	-0.725940000	0.000000000
C	-1.235102000	-1.406546000	0.000000000
C	0.000000000	-0.726320000	0.000000000
C	0.000000000	0.726320000	0.000000000
H	-1.235274000	2.494145000	0.000000000
H	-1.235274000	-2.494145000	0.000000000
C	1.235102000	1.406546000	0.000000000
C	2.450052000	0.725940000	0.000000000
C	2.450052000	-0.725940000	0.000000000
C	1.235102000	-1.406546000	0.000000000
H	1.235274000	-2.494145000	0.000000000
H	1.235274000	2.494145000	0.000000000
C	-3.710418000	1.409129000	0.000000000
C	-4.887920000	0.715140000	0.000000000
H	-5.834602000	1.247232000	0.000000000
C	-4.887920000	-0.715140000	0.000000000
H	-5.834602000	-1.247232000	0.000000000
C	-3.710418000	-1.409129000	0.000000000
H	-3.708863000	-2.495980000	0.000000000
H	-3.708863000	2.495980000	0.000000000
C	3.710418000	1.409129000	0.000000000
C	4.887920000	0.715140000	0.000000000
H	5.834602000	1.247232000	0.000000000
C	4.887920000	-0.715140000	0.000000000
H	5.834602000	-1.247232000	0.000000000
C	3.710418000	-1.409129000	0.000000000
H	3.708863000	-2.495980000	0.000000000
H	3.708863000	2.495980000	0.000000000

Pentacene

C	-2.467115000	1.408070000	0.000000000
C	-3.677901000	0.727630000	0.000000000
C	-3.677901000	-0.727630000	0.000000000
C	-2.467115000	-1.408070000	0.000000000
C	-1.226237000	-0.728620000	0.000000000
C	-1.226237000	0.728620000	0.000000000
H	-2.467403000	2.495622000	0.000000000
H	-2.467403000	-2.495622000	0.000000000
C	0.000000000	1.408716000	0.000000000
C	1.226237000	0.728620000	0.000000000
C	1.226237000	-0.728620000	0.000000000
C	0.000000000	-1.408716000	0.000000000
H	0.000000000	-2.496160000	0.000000000
H	0.000000000	2.496160000	0.000000000
C	-4.940835000	1.410334000	0.000000000
C	-6.116597000	0.716377000	0.000000000
H	-7.063938000	1.247277000	0.000000000
C	-6.116597000	-0.716377000	0.000000000
H	-7.063938000	-1.247277000	0.000000000
C	-4.940835000	-1.410334000	0.000000000
H	-4.939202000	-2.497168000	0.000000000
H	-4.939202000	2.497168000	0.000000000
C	2.467115000	1.408070000	0.000000000
C	3.677901000	0.727630000	0.000000000
C	3.677901000	-0.727630000	0.000000000
C	2.467115000	-1.408070000	0.000000000
H	2.467403000	-2.495622000	0.000000000
H	2.467403000	2.495622000	0.000000000
C	4.940835000	1.410334000	0.000000000
C	6.116597000	0.716377000	0.000000000
H	7.063938000	1.247277000	0.000000000
C	6.116597000	-0.716377000	0.000000000
H	7.063938000	-1.247277000	0.000000000
C	4.940835000	-1.410334000	0.000000000
H	4.939202000	-2.497168000	0.000000000

H	4.939202000	2.497168000	0.000000000

Hexacene

C	-1.231952000	-1.409933000	0.000000000
C	0.000000000	-0.730384000	0.000000000
C	0.000000000	0.730384000	0.000000000
C	-1.231952000	1.409933000	0.000000000
C	-2.456833000	0.729293000	0.000000000
C	-2.456833000	-0.729293000	0.000000000
H	-1.232043000	-2.497180000	0.000000000
H	-1.232043000	2.497180000	0.000000000
C	1.231952000	1.409933000	0.000000000
C	1.231952000	-1.409933000	0.000000000
C	2.456833000	0.729293000	0.000000000
C	2.456833000	-0.729293000	0.000000000
H	1.232043000	2.497180000	0.000000000
H	1.232043000	-2.497180000	0.000000000
C	3.697917000	1.408269000	0.000000000
C	-3.697917000	1.408269000	0.000000000
C	-3.697917000	-1.408269000	0.000000000
C	3.697917000	-1.408269000	0.000000000
C	4.910181000	-0.727270000	0.000000000
C	4.910181000	0.727270000	0.000000000
C	-4.910181000	0.727270000	0.000000000
C	-4.910181000	-0.727270000	0.000000000
H	3.697987000	2.495673000	0.000000000
H	3.697987000	-2.495673000	0.000000000
H	-3.697987000	-2.495673000	0.000000000
H	-3.697987000	2.495673000	0.000000000
C	6.172004000	1.409887000	0.000000000
C	6.172004000	-1.409887000	0.000000000
C	-6.172004000	1.409887000	0.000000000
C	-6.172004000	-1.409887000	0.000000000
C	7.348874000	-0.715869000	0.000000000
H	8.296042000	-1.246838000	0.000000000
C	7.348874000	0.715869000	0.000000000
H	8.296042000	1.246838000	0.000000000
C	-7.348874000	0.715869000	0.000000000
H	-8.296042000	1.246838000	0.000000000
C	-7.348874000	-0.715869000	0.000000000
H	-8.296042000	-1.246838000	0.000000000
H	6.170020000	2.496622000	0.000000000
H	6.170020000	-2.496622000	0.000000000
H	-6.170020000	-2.496622000	0.000000000
H	-6.170020000	2.496622000	0.000000000

Heptacene

C	-2.462501000	-1.409493000	0.000000000
C	-1.232676000	-0.731134000	0.000000000
C	-1.232676000	0.731134000	0.000000000
C	-2.462501000	1.409493000	0.000000000
C	-3.694602000	0.728336000	0.000000000
C	-3.694602000	-0.728336000	0.000000000
H	-2.463073000	-2.496833000	0.000000000
H	-2.463073000	2.496833000	0.000000000
C	0.000000000	1.410542000	0.000000000
C	0.000000000	-1.410542000	0.000000000
C	1.232676000	0.731134000	0.000000000
C	1.232676000	-0.731134000	0.000000000
H	0.000000000	2.497838000	0.000000000
H	0.000000000	-2.497838000	0.000000000
C	2.462501000	1.409493000	0.000000000
C	-4.929474000	1.406798000	0.000000000
C	-4.929474000	-1.406798000	0.000000000

C	2.462501000	-1.409493000	0.000000000
C	3.694602000	-0.728336000	0.000000000
C	3.694602000	0.728336000	0.000000000
C	-6.148980000	0.724962000	0.000000000
C	-6.148980000	-0.724962000	0.000000000
H	2.463073000	2.496833000	0.000000000
H	2.463073000	-2.496833000	0.000000000
H	-4.930037000	-2.494323000	0.000000000
H	-4.930037000	2.494323000	0.000000000
C	4.929474000	1.406798000	0.000000000
C	4.929474000	-1.406798000	0.000000000
C	-7.405949000	1.407943000	0.000000000
C	-7.405949000	-1.407943000	0.000000000
C	6.148980000	-0.724962000	0.000000000
C	6.148980000	0.724962000	0.000000000
C	-8.586649000	0.713612000	0.000000000
H	-9.532928000	1.246385000	0.000000000
C	-8.586649000	-0.713612000	0.000000000
H	-9.532928000	-1.246385000	0.000000000
H	4.930037000	2.494323000	0.000000000
H	4.930037000	-2.494323000	0.000000000
H	-7.403876000	-2.494802000	0.000000000
H	-7.403876000	2.494802000	0.000000000
C	7.405949000	1.407943000	0.000000000
C	7.405949000	-1.407943000	0.000000000
C	8.586649000	-0.713612000	0.000000000
H	9.532928000	-1.246385000	0.000000000
C	8.586649000	0.713612000	0.000000000
H	9.532928000	1.246385000	0.000000000
H	7.403876000	2.494802000	0.000000000
H	7.403876000	-2.494802000	0.000000000

Octacene

C	0.000000000	6.161556000	1.406192000
C	0.000000000	7.383168000	0.724214000
C	0.000000000	7.383168000	-0.724214000
C	0.000000000	6.161556000	-1.406192000
C	0.000000000	4.929265000	-0.727599000
C	0.000000000	4.929265000	0.727599000
H	0.000000000	6.161964000	2.493726000
H	0.000000000	6.161964000	-2.493726000
C	0.000000000	3.693584000	1.408861000
C	0.000000000	2.467292000	0.730743000
C	0.000000000	2.467292000	-0.730743000
C	0.000000000	3.693584000	-1.408861000
H	0.000000000	3.694022000	-2.496223000
H	0.000000000	3.694022000	2.496223000
C	0.000000000	8.638847000	1.407404000
C	0.000000000	9.820440000	0.713036000
H	0.000000000	10.766518000	1.246165000
C	0.000000000	9.820440000	-0.713036000
H	0.000000000	10.766518000	-1.246165000
C	0.000000000	8.638847000	-1.407404000
H	0.000000000	8.636642000	-2.494266000
H	0.000000000	8.636642000	2.494266000
C	0.000000000	1.230586000	1.410189000
C	0.000000000	0.000000000	0.731837000
C	0.000000000	0.000000000	-0.731837000
C	0.000000000	1.230586000	-1.410189000
H	0.000000000	1.230693000	-2.497503000
H	0.000000000	1.230693000	2.497503000
C	0.000000000	-1.230586000	1.410189000
C	0.000000000	-2.467292000	0.730743000
C	0.000000000	-2.467292000	-0.730743000
C	0.000000000	-1.230586000	-1.410189000
H	0.000000000	-1.230693000	-2.497503000
H	0.000000000	-1.230693000	2.497503000

C	0.000000000	-3.693584000	1.408861000
C	0.000000000	-4.929265000	0.727599000
C	0.000000000	-4.929265000	-0.727599000
C	0.000000000	-3.693584000	-1.408861000
H	0.000000000	-3.694022000	-2.496223000
H	0.000000000	-3.694022000	2.496223000
C	0.000000000	-6.161556000	1.406192000
C	0.000000000	-7.383168000	0.724214000
C	0.000000000	-7.383168000	-0.724214000
C	0.000000000	-6.161556000	-1.406192000
H	0.000000000	-6.161964000	-2.493726000
H	0.000000000	-6.161964000	2.493726000
C	0.000000000	-8.638847000	1.407404000
C	0.000000000	-9.820440000	0.713036000
H	0.000000000	-10.766518000	1.246165000
C	0.000000000	-9.820440000	-0.713036000
H	0.000000000	-10.766518000	-1.246165000
H	0.000000000	-8.636642000	2.494266000
C	0.000000000	-8.638847000	-1.407404000
H	0.000000000	-8.636642000	-2.494266000

Nonacene

C	0.000000000	7.393599000	1.406034000
C	0.000000000	8.615387000	0.724105000
C	0.000000000	8.615387000	-0.724105000
C	0.000000000	7.393599000	-1.406034000
C	0.000000000	6.161941000	-0.727299000
C	0.000000000	6.161941000	0.727299000
H	0.000000000	7.393911000	2.493574000
H	0.000000000	7.393911000	-2.493574000
C	0.000000000	4.925163000	1.408521000
C	0.000000000	3.700853000	0.730322000
C	0.000000000	3.700853000	-0.730322000
C	0.000000000	4.925163000	-1.408521000
H	0.000000000	4.925456000	-2.495902000
H	0.000000000	4.925456000	2.495902000
C	0.000000000	9.871002000	1.407369000
C	0.000000000	11.052566000	0.713016000
H	0.000000000	11.998648000	1.246140000
C	0.000000000	11.052566000	-0.713016000
H	0.000000000	11.998648000	-1.246140000
C	0.000000000	9.871002000	-1.407369000
H	0.000000000	9.868764000	-2.494231000
H	0.000000000	9.868764000	2.494231000
C	0.000000000	2.461417000	1.409726000
C	0.000000000	1.234219000	0.731689000
C	0.000000000	1.234219000	-0.731689000
C	0.000000000	2.461417000	-1.409726000
H	0.000000000	2.461408000	-2.497071000
H	0.000000000	2.461408000	2.497071000
C	0.000000000	0.000000000	1.409970000
C	0.000000000	-1.234219000	0.731689000
C	0.000000000	-1.234219000	-0.731689000
C	0.000000000	0.000000000	-1.409970000
H	0.000000000	0.000000000	-2.497311000
H	0.000000000	0.000000000	2.497311000
C	0.000000000	-2.461417000	1.409726000
C	0.000000000	-3.700853000	0.730322000
C	0.000000000	-3.700853000	-0.730322000
C	0.000000000	-2.461417000	-1.409726000
H	0.000000000	-2.461408000	-2.497071000
H	0.000000000	-2.461408000	2.497071000
C	0.000000000	-4.925163000	1.408521000
C	0.000000000	-6.161941000	0.727299000
C	0.000000000	-6.161941000	-0.727299000
C	0.000000000	-4.925163000	-1.408521000
H	0.000000000	-4.925456000	-2.495902000

H	0.000000000	-4.925456000	2.495902000
C	0.000000000	-7.393599000	1.406034000
C	0.000000000	-8.615387000	0.724105000
C	0.000000000	-8.615387000	-0.724105000
H	0.000000000	-7.393911000	2.493574000
C	0.000000000	-7.393599000	-1.406034000
H	0.000000000	-7.393911000	-2.493574000
C	0.000000000	-9.871002000	1.407369000
C	0.000000000	-11.052566000	0.713016000
H	0.000000000	-11.998648000	1.246140000
C	0.000000000	-11.052566000	-0.713016000
H	0.000000000	-11.998648000	-1.246140000
C	0.000000000	-9.871002000	-1.407369000
H	0.000000000	-9.868764000	-2.494231000
H	0.000000000	-9.868764000	2.494231000

Decacene

C	0.000000000	8.625482000	1.406071000
C	0.000000000	9.846838000	0.724216000
C	0.000000000	9.846838000	-0.724216000
C	0.000000000	8.625482000	-1.406071000
C	0.000000000	7.393632000	-0.727249000
C	0.000000000	7.393632000	0.727249000
H	0.000000000	8.625742000	2.493611000
H	0.000000000	8.625742000	-2.493611000
C	0.000000000	6.156961000	1.408424000
C	0.000000000	4.933261000	0.730088000
C	0.000000000	4.933261000	-0.730088000
C	0.000000000	6.156961000	-1.408424000
H	0.000000000	6.157157000	-2.495812000
H	0.000000000	6.157157000	2.495812000
C	0.000000000	11.102750000	1.407488000
C	0.000000000	12.284046000	0.713158000
H	0.000000000	13.230190000	1.246171000
C	0.000000000	12.284046000	-0.713158000
H	0.000000000	13.230190000	-1.246171000
C	0.000000000	11.102750000	-1.407488000
H	0.000000000	11.100514000	-2.494348000
H	0.000000000	11.100514000	2.494348000
C	0.000000000	3.692774000	1.409476000
C	0.000000000	2.467690000	0.731370000
C	0.000000000	2.467690000	-0.731370000
C	0.000000000	3.692774000	-1.409476000
H	0.000000000	3.692625000	-2.496840000
H	0.000000000	3.692625000	2.496840000
C	0.000000000	1.230735000	1.409592000
C	0.000000000	0.000000000	0.731651000
C	0.000000000	0.000000000	-0.731651000
C	0.000000000	1.230735000	-1.409592000
H	0.000000000	1.230618000	-2.496964000
H	0.000000000	1.230618000	2.496964000
C	0.000000000	-1.230735000	1.409592000
C	0.000000000	-2.467690000	0.731370000
C	0.000000000	-2.467690000	-0.731370000
C	0.000000000	-1.230735000	-1.409592000
H	0.000000000	-1.230618000	-2.496964000
H	0.000000000	-1.230618000	2.496964000
C	0.000000000	-3.692774000	1.409476000
C	0.000000000	-4.933261000	0.730088000
C	0.000000000	-4.933261000	-0.730088000
C	0.000000000	-3.692774000	-1.409476000
H	0.000000000	-3.692625000	-2.496840000
H	0.000000000	-3.692625000	2.496840000
C	0.000000000	-6.156961000	1.408424000
C	0.000000000	-7.393632000	0.727249000
C	0.000000000	-7.393632000	-0.727249000
H	0.000000000	-6.157157000	2.495812000

C	0.000000000	-6.156961000	-1.408424000
H	0.000000000	-6.157157000	-2.495812000
C	0.000000000	-8.625482000	1.406071000
C	0.000000000	-9.846838000	0.724216000
C	0.000000000	-9.846838000	-0.724216000
C	0.000000000	-8.625482000	-1.406071000
H	0.000000000	-8.625742000	-2.493611000
H	0.000000000	-8.625742000	2.493611000
C	0.000000000	-11.102750000	1.407488000
C	0.000000000	-12.284046000	0.713158000
H	0.000000000	-13.230190000	1.246171000
C	0.000000000	-12.284046000	-0.713158000
H	0.000000000	-13.230190000	-1.246171000
C	0.000000000	-11.102750000	-1.407488000
H	0.000000000	-11.100514000	-2.494348000
H	0.000000000	-11.100514000	2.494348000

Undecacene

C	0.000000000	9.856685000	1.406257000
C	0.000000000	11.077690000	0.724349000
C	0.000000000	11.077690000	-0.724349000
C	0.000000000	9.856685000	-1.406257000
C	0.000000000	8.624666000	-0.727394000
C	0.000000000	8.624666000	0.727394000
H	0.000000000	9.857828000	2.493797000
H	0.000000000	9.857828000	-2.493797000
C	0.000000000	7.388442000	1.408521000
C	0.000000000	6.164712000	0.730160000
C	0.000000000	6.164712000	-0.730160000
C	0.000000000	7.388442000	-1.408521000
H	0.000000000	7.389712000	-2.495927000
H	0.000000000	7.389712000	2.495927000
C	0.000000000	12.333683000	1.407616000
C	0.000000000	13.514866000	0.713188000
H	0.000000000	14.460799000	1.246585000
C	0.000000000	13.514866000	-0.713188000
H	0.000000000	14.460799000	-1.246585000
C	0.000000000	12.333683000	-1.407616000
H	0.000000000	12.332048000	-2.494469000
H	0.000000000	12.332048000	2.494469000
C	0.000000000	4.924304000	1.409452000
C	0.000000000	3.699945000	0.731307000
C	0.000000000	3.699945000	-0.731307000
C	0.000000000	4.924304000	-1.409452000
H	0.000000000	4.925084000	-2.496851000
H	0.000000000	4.925084000	2.496851000
C	0.000000000	2.461950000	1.409396000
C	0.000000000	1.233406000	0.731488000
C	0.000000000	1.233406000	-0.731488000
C	0.000000000	2.461950000	-1.409396000
H	0.000000000	2.462281000	-2.496814000
H	0.000000000	2.462281000	2.496814000
C	0.000000000	0.000000000	1.409253000
C	0.000000000	-1.233406000	0.731488000
C	0.000000000	-1.233406000	-0.731488000
C	0.000000000	0.000000000	-1.409253000
H	0.000000000	0.000000000	-2.496679000
H	0.000000000	0.000000000	2.496679000
C	0.000000000	-2.461950000	1.409396000
C	0.000000000	-3.699945000	0.731307000
C	0.000000000	-3.699945000	-0.731307000
C	0.000000000	-2.461950000	-1.409396000
H	0.000000000	-2.462281000	-2.496814000
H	0.000000000	-2.462281000	2.496814000
C	0.000000000	-4.924304000	1.409452000
C	0.000000000	-6.164712000	0.730160000
C	0.000000000	-6.164712000	-0.730160000

H	0.000000000	-4.925084000	2.496851000
C	0.000000000	-4.924304000	-1.409452000
H	0.000000000	-4.925084000	-2.496851000
C	0.000000000	-7.388442000	1.408521000
C	0.000000000	-8.624666000	0.727394000
C	0.000000000	-8.624666000	-0.727394000
C	0.000000000	-7.388442000	-1.408521000
H	0.000000000	-7.389712000	-2.495927000
H	0.000000000	-7.389712000	2.495927000
C	0.000000000	-9.856685000	1.406257000
C	0.000000000	-11.077690000	0.724349000
C	0.000000000	-11.077690000	-0.724349000
C	0.000000000	-9.856685000	-1.406257000
H	0.000000000	-9.857828000	-2.493797000
H	0.000000000	-9.857828000	2.493797000
C	0.000000000	-12.333683000	1.407616000
C	0.000000000	-13.514866000	0.713188000
H	0.000000000	-14.460799000	1.246585000
C	0.000000000	-13.514866000	-0.713188000
H	0.000000000	-14.460799000	-1.246585000
C	0.000000000	-12.333683000	-1.407616000
H	0.000000000	-12.332048000	-2.494469000
H	0.000000000	-12.332048000	2.494469000

Dodecacene

C	0.000000000	11.088588000	1.406307000
C	0.000000000	12.309248000	0.724453000
C	0.000000000	12.309248000	-0.724453000
C	0.000000000	11.088588000	-1.406307000
C	0.000000000	9.856194000	-0.727433000
C	0.000000000	9.856194000	0.727433000
H	0.000000000	11.089546000	2.493845000
H	0.000000000	11.089546000	-2.493845000
C	0.000000000	8.620398000	1.408563000
C	0.000000000	7.396352000	0.730132000
C	0.000000000	7.396352000	-0.730132000
C	0.000000000	8.620398000	-1.408563000
H	0.000000000	8.621513000	-2.495962000
H	0.000000000	8.621513000	2.495962000
C	0.000000000	13.565501000	1.407696000
C	0.000000000	14.746501000	0.713309000
H	0.000000000	15.692535000	1.246525000
C	0.000000000	14.746501000	-0.713309000
H	0.000000000	15.692535000	-1.246525000
C	0.000000000	13.565501000	-1.407696000
H	0.000000000	13.563743000	-2.494550000
H	0.000000000	13.563743000	2.494550000
C	0.000000000	6.156238000	1.409470000
C	0.000000000	4.931890000	0.731190000
C	0.000000000	4.931890000	-0.731190000
C	0.000000000	6.156238000	-1.409470000
H	0.000000000	6.156974000	-2.496863000
H	0.000000000	6.156974000	2.496863000
C	0.000000000	3.693684000	1.409320000
C	0.000000000	2.465943000	0.731231000
C	0.000000000	2.465943000	-0.731231000
C	0.000000000	3.693684000	-1.409320000
H	0.000000000	3.694078000	-2.496739000
H	0.000000000	3.694078000	2.496739000
C	0.000000000	1.231270000	1.409001000
C	0.000000000	0.000000000	0.731130000
C	0.000000000	0.000000000	-0.731130000
C	0.000000000	1.231270000	-1.409001000
H	0.000000000	1.231392000	-2.496438000
H	0.000000000	1.231392000	2.496438000
C	0.000000000	-1.231270000	1.409001000
C	0.000000000	-2.465943000	0.731231000

C	0.000000000	-2.465943000	-0.731231000
C	0.000000000	-1.231270000	-1.409001000
H	0.000000000	-1.231392000	-2.496438000
H	0.000000000	-1.231392000	2.496438000
C	0.000000000	-3.693684000	1.409320000
C	0.000000000	-4.931890000	0.731190000
C	0.000000000	-4.931890000	-0.731190000
H	0.000000000	-3.694078000	2.496739000
C	0.000000000	-3.693684000	-1.409320000
H	0.000000000	-3.694078000	-2.496739000
C	0.000000000	-6.156238000	1.409470000
C	0.000000000	-7.396352000	0.730132000
C	0.000000000	-7.396352000	-0.730132000
C	0.000000000	-6.156238000	-1.409470000
H	0.000000000	-6.156974000	-2.496863000
H	0.000000000	-6.156974000	2.496863000
C	0.000000000	-8.620398000	1.408563000
C	0.000000000	-9.856194000	0.727433000
C	0.000000000	-9.856194000	-0.727433000
C	0.000000000	-8.620398000	-1.408563000
H	0.000000000	-8.621513000	-2.495962000
H	0.000000000	-8.621513000	2.495962000
C	0.000000000	-11.088588000	1.406307000
C	0.000000000	-12.309248000	0.724453000
C	0.000000000	-12.309248000	-0.724453000
C	0.000000000	-11.088588000	-1.406307000
H	0.000000000	-11.089546000	-2.493845000
H	0.000000000	-11.089546000	2.493845000
C	0.000000000	-13.565501000	1.407696000
C	0.000000000	-14.746501000	0.713309000
H	0.000000000	-15.692535000	1.246525000
C	0.000000000	-14.746501000	-0.713309000
H	0.000000000	-15.692535000	-1.246525000
C	0.000000000	-13.565501000	-1.407696000
H	0.000000000	-13.563743000	-2.494550000
H	0.000000000	-13.563743000	2.494550000

Triplet geometries (B3LYP/6-31G**)

Napthalene

C	-1.237876000	1.401369000	0.000000000
C	-2.486974000	0.681592000	0.000000000
C	-2.486974000	-0.681592000	0.000000000
C	-1.237876000	-1.401369000	0.000000000
C	0.000000000	-0.725032000	0.000000000
C	0.000000000	0.725032000	0.000000000
H	-1.245144000	2.487413000	0.000000000
H	-3.418707000	1.238706000	0.000000000
H	-3.418707000	-1.238706000	0.000000000
H	-1.245144000	-2.487413000	0.000000000
C	1.237876000	1.401369000	0.000000000
C	2.486974000	0.681592000	0.000000000
H	3.418707000	1.238706000	0.000000000
C	2.486974000	-0.681592000	0.000000000
H	3.418707000	-1.238706000	0.000000000
C	1.237876000	-1.401369000	0.000000000
H	1.245144000	-2.487413000	0.000000000
H	1.245144000	2.487413000	0.000000000

Anthracene

C	0.000000000	1.406685000	0.000000000
C	-1.254468000	0.720579000	0.000000000
C	-1.254468000	-0.720579000	0.000000000

C	0.000000000	-1.406685000	0.000000000
C	1.254468000	-0.720579000	0.000000000
C	1.254468000	0.720579000	0.000000000
H	0.000000000	2.493609000	0.000000000
H	0.000000000	-2.493609000	0.000000000
C	2.479591000	1.395892000	0.000000000
C	3.704323000	0.691547000	0.000000000
H	4.639806000	1.242492000	0.000000000
C	3.704323000	-0.691547000	0.000000000
H	4.639806000	-1.242492000	0.000000000
C	2.479591000	-1.395892000	0.000000000
H	2.482973000	-2.482681000	0.000000000
H	2.482973000	2.482681000	0.000000000
C	-2.479591000	1.395892000	0.000000000
C	-3.704323000	0.691547000	0.000000000
H	-4.639806000	1.242492000	0.000000000
C	-3.704323000	-0.691547000	0.000000000
H	-4.639806000	-1.242492000	0.000000000
C	-2.479591000	-1.395892000	0.000000000
H	-2.482973000	-2.482681000	0.000000000
H	-2.482973000	2.482681000	0.000000000

Tetracene

C	-1.230778000	1.405106000	0.000000000
C	-2.485893000	0.717443000	0.000000000
C	-2.485893000	-0.717443000	0.000000000
C	-1.230778000	-1.405106000	0.000000000
C	0.000000000	-0.730888000	0.000000000
C	0.000000000	0.730888000	0.000000000
H	-1.234930000	2.492377000	0.000000000
H	-1.234930000	-2.492377000	0.000000000
C	1.230778000	1.405106000	0.000000000
C	2.485893000	0.717443000	0.000000000
C	2.485893000	-0.717443000	0.000000000
C	1.230778000	-1.405106000	0.000000000
H	1.234930000	-2.492377000	0.000000000
H	1.234930000	2.492377000	0.000000000
C	-3.715408000	1.397431000	0.000000000
C	-4.924323000	0.698580000	0.000000000
H	-5.863540000	1.243371000	0.000000000
C	-4.924323000	-0.698580000	0.000000000
H	-5.863540000	-1.243371000	0.000000000
C	-3.715408000	-1.397431000	0.000000000
H	-3.715321000	-2.484342000	0.000000000
H	-3.715321000	2.484342000	0.000000000
C	3.715408000	1.397431000	0.000000000
C	4.924323000	0.698580000	0.000000000
H	5.863540000	1.243371000	0.000000000
C	4.924323000	-0.698580000	0.000000000
H	5.863540000	-1.243371000	0.000000000
C	3.715408000	-1.397431000	0.000000000
H	3.715321000	-2.484342000	0.000000000
H	3.715321000	2.484342000	0.000000000

Pentacene

C	-2.463400000	1.403296000	0.000000000
C	-3.711875000	0.717599000	0.000000000
C	-3.711875000	-0.717599000	0.000000000
C	-2.463400000	-1.403296000	0.000000000
C	-1.243847000	-0.729750000	0.000000000
C	-1.243847000	0.729750000	0.000000000
H	-2.466997000	2.490804000	0.000000000
H	-2.466997000	-2.490804000	0.000000000
C	0.000000000	1.408663000	0.000000000

C	1.243847000	0.729750000	0.000000000
C	1.243847000	-0.729750000	0.000000000
C	0.000000000	-1.408663000	0.000000000
H	0.000000000	-2.495882000	0.000000000
H	0.000000000	2.495882000	0.000000000
C	-4.948354000	1.400039000	0.000000000
C	-6.147039000	0.703490000	0.000000000
H	-7.088690000	1.244209000	0.000000000
C	-6.147039000	-0.703490000	0.000000000
H	-7.088690000	-1.244209000	0.000000000
C	-4.948354000	-1.400039000	0.000000000
H	-4.946939000	-2.486960000	0.000000000
H	-4.946939000	2.486960000	0.000000000
C	2.463400000	1.403296000	0.000000000
C	3.711875000	0.717599000	0.000000000
C	3.711875000	-0.717599000	0.000000000
C	2.463400000	-1.403296000	0.000000000
H	2.466997000	-2.490804000	0.000000000
H	2.466997000	2.490804000	0.000000000
C	4.948354000	1.400039000	0.000000000
C	6.147039000	0.703490000	0.000000000
H	7.088690000	1.244209000	0.000000000
C	6.147039000	-0.703490000	0.000000000
H	7.088690000	-1.244209000	0.000000000
C	4.948354000	-1.400039000	0.000000000
H	4.946939000	-2.486960000	0.000000000
H	4.946939000	2.486960000	0.000000000

Hexacene

C	-1.228908000	-1.408358000	0.000000000
C	0.000000000	-0.733717000	0.000000000
C	0.000000000	0.733717000	0.000000000
C	-1.228908000	1.408358000	0.000000000
C	-2.478565000	0.727395000	0.000000000
C	-2.478565000	-0.727395000	0.000000000
H	-1.230957000	-2.495702000	0.000000000
H	-1.230957000	2.495702000	0.000000000
C	1.228908000	1.408358000	0.000000000
C	1.228908000	-1.408358000	0.000000000
C	2.478565000	0.727395000	0.000000000
C	2.478565000	-0.727395000	0.000000000
H	1.230957000	2.495702000	0.000000000
H	1.230957000	-2.495702000	0.000000000
C	3.696331000	1.402995000	0.000000000
C	-3.696331000	1.402995000	0.000000000
C	-3.696331000	-1.402995000	0.000000000
C	3.696331000	-1.402995000	0.000000000
C	4.936789000	-0.718992000	0.000000000
C	4.936789000	0.718992000	0.000000000
C	-4.936789000	0.718992000	0.000000000
C	-4.936789000	-0.718992000	0.000000000
H	3.698642000	2.490584000	0.000000000
H	3.698642000	-2.490584000	0.000000000
H	-3.698642000	-2.490584000	0.000000000
H	-3.698642000	2.490584000	0.000000000
C	6.179623000	1.402409000	0.000000000
C	6.179623000	-1.402409000	0.000000000
C	-6.179623000	1.402409000	0.000000000
C	-6.179623000	-1.402409000	0.000000000
C	7.371505000	-0.706982000	0.000000000
H	8.314819000	-1.244877000	0.000000000
C	7.371505000	0.706982000	0.000000000
H	8.314819000	1.244877000	0.000000000
C	-7.371505000	0.706982000	0.000000000
H	-8.314819000	1.244877000	0.000000000
C	-7.371505000	-0.706982000	0.000000000
H	-8.314819000	-1.244877000	0.000000000

H	6.177691000	2.489313000	0.000000000
H	6.177691000	-2.489313000	0.000000000
H	-6.177691000	-2.489313000	0.000000000
H	-6.177691000	2.489313000	0.000000000

Heptacene

C	-2.459057000	-1.407579000	0.000000000
C	-1.239478000	-0.733171000	0.000000000
C	-1.239478000	0.733171000	0.000000000
C	-2.459057000	1.407579000	0.000000000
C	-3.708456000	0.726187000	0.000000000
C	-3.708456000	-0.726187000	0.000000000
H	-2.461222000	-2.495013000	0.000000000
H	-2.461222000	2.495013000	0.000000000
C	0.000000000	1.409880000	0.000000000
C	0.000000000	-1.409880000	0.000000000
C	1.239478000	0.733171000	0.000000000
C	1.239478000	-0.733171000	0.000000000
H	0.000000000	2.497235000	0.000000000
H	0.000000000	-2.497235000	0.000000000
C	2.459057000	1.407579000	0.000000000
C	-4.928671000	1.403509000	0.000000000
C	-4.928671000	-1.403509000	0.000000000
C	2.459057000	-1.407579000	0.000000000
C	3.708456000	-0.726187000	0.000000000
C	3.708456000	0.726187000	0.000000000
C	-6.161949000	0.720657000	0.000000000
C	-6.161949000	-0.720657000	0.000000000
H	2.461222000	2.495013000	0.000000000
H	2.461222000	-2.495013000	0.000000000
H	-4.930041000	-2.491118000	0.000000000
H	-4.930041000	2.491118000	0.000000000
C	4.928671000	1.403509000	0.000000000
C	4.928671000	-1.403509000	0.000000000
C	-7.409907000	1.404353000	0.000000000
C	-7.409907000	-1.404353000	0.000000000
C	6.161949000	-0.720657000	0.000000000
C	6.161949000	0.720657000	0.000000000
C	-8.597105000	0.709524000	0.000000000
H	-9.541585000	1.245412000	0.000000000
C	-8.597105000	-0.709524000	0.000000000
H	-9.541585000	-1.245412000	0.000000000
H	4.930041000	2.491118000	0.000000000
H	4.930041000	-2.491118000	0.000000000
H	-7.407779000	-2.491239000	0.000000000
H	-7.407779000	2.491239000	0.000000000
C	7.409907000	1.404353000	0.000000000
C	7.409907000	-1.404353000	0.000000000
C	8.597105000	-0.709524000	0.000000000
H	9.541585000	-1.245412000	0.000000000
C	8.597105000	0.709524000	0.000000000
H	9.541585000	1.245412000	0.000000000
H	7.407779000	2.491239000	0.000000000
H	7.407779000	-2.491239000	0.000000000

Octacene

C	0.000000000	6.160354000	1.404307000
C	0.000000000	7.387792000	0.722222000
C	0.000000000	7.387792000	-0.722222000
C	0.000000000	6.160354000	-1.404307000
C	0.000000000	4.936305000	-0.725939000
C	0.000000000	4.936305000	0.725939000
H	0.000000000	6.161187000	2.491907000
H	0.000000000	6.161187000	-2.491907000

C	0.000000000	3.690156000	1.407200000
C	0.000000000	2.474156000	0.731628000
C	0.000000000	2.474156000	-0.731628000
C	0.000000000	3.690156000	-1.407200000
H	0.000000000	3.691832000	-2.494667000
H	0.000000000	3.691832000	2.494667000
C	0.000000000	8.639740000	1.405896000
C	0.000000000	9.823635000	0.711406000
H	0.000000000	10.768950000	1.245846000
C	0.000000000	9.823635000	-0.711406000
H	0.000000000	10.768950000	-1.245846000
C	0.000000000	8.639740000	-1.405896000
H	0.000000000	8.637611000	-2.492764000
H	0.000000000	8.637611000	2.492764000
C	0.000000000	1.228342000	1.409844000
C	0.000000000	0.000000000	0.734986000
C	0.000000000	0.000000000	-0.734986000
C	0.000000000	1.228342000	-1.409844000
H	0.000000000	1.229426000	-2.497242000
H	0.000000000	1.229426000	2.497242000
C	0.000000000	-1.228342000	1.409844000
C	0.000000000	-2.474156000	0.731628000
C	0.000000000	-2.474156000	-0.731628000
C	0.000000000	-1.228342000	-1.409844000
H	0.000000000	-1.229426000	-2.497242000
H	0.000000000	-1.229426000	2.497242000
C	0.000000000	-3.690156000	1.407200000
C	0.000000000	-4.936305000	0.725939000
C	0.000000000	-4.936305000	-0.725939000
C	0.000000000	-3.690156000	-1.407200000
H	0.000000000	-3.691832000	-2.494667000
H	0.000000000	-3.691832000	2.494667000
C	0.000000000	-6.160354000	1.404307000
C	0.000000000	-7.387792000	0.722222000
C	0.000000000	-7.387792000	-0.722222000
C	0.000000000	-6.160354000	-1.404307000
H	0.000000000	-6.161187000	-2.491907000
H	0.000000000	-6.161187000	2.491907000
C	0.000000000	-8.639740000	1.405896000
C	0.000000000	-9.823635000	0.711406000
H	0.000000000	-10.768950000	1.245846000
C	0.000000000	-9.823635000	-0.711406000
H	0.000000000	-10.768950000	-1.245846000
H	0.000000000	-8.637611000	2.492764000
C	0.000000000	-8.639740000	-1.405896000
H	0.000000000	-8.637611000	-2.492764000

Nonacene

C	0.000000000	7.391373000	1.405158000
C	0.000000000	8.614196000	0.723594000
C	0.000000000	8.614196000	-0.723594000
C	0.000000000	7.391373000	-1.405158000
C	0.000000000	6.163398000	-0.726241000
C	0.000000000	6.163398000	0.726241000
H	0.000000000	7.391896000	2.492744000
H	0.000000000	7.391896000	-2.492744000
C	0.000000000	4.921492000	1.407254000
C	0.000000000	3.705440000	0.730442000
C	0.000000000	3.705440000	-0.730442000
C	0.000000000	4.921492000	-1.407254000
H	0.000000000	4.922651000	-2.494725000
H	0.000000000	4.922651000	2.494725000
C	0.000000000	9.869210000	1.407121000
C	0.000000000	11.050702000	0.712824000
H	0.000000000	11.996631000	1.246190000
C	0.000000000	11.050702000	-0.712824000
H	0.000000000	11.996631000	-1.246190000

C	0.000000000	9.869210000	-1.407121000
H	0.000000000	9.867126000	-2.493975000
H	0.000000000	9.867126000	2.493975000
C	0.000000000	2.457593000	1.409444000
C	0.000000000	1.237112000	0.734615000
C	0.000000000	1.237112000	-0.734615000
C	0.000000000	2.457593000	-1.409444000
H	0.000000000	2.458891000	-2.496878000
H	0.000000000	2.458891000	2.496878000
C	0.000000000	0.000000000	1.410624000
C	0.000000000	-1.237112000	0.734615000
C	0.000000000	-1.237112000	-0.734615000
C	0.000000000	0.000000000	-1.410624000
H	0.000000000	0.000000000	-2.498027000
H	0.000000000	0.000000000	2.498027000
C	0.000000000	-2.457593000	1.409444000
C	0.000000000	-3.705440000	0.730442000
C	0.000000000	-3.705440000	-0.730442000
C	0.000000000	-2.457593000	-1.409444000
H	0.000000000	-2.458891000	-2.496878000
H	0.000000000	-2.458891000	2.496878000
C	0.000000000	-4.921492000	1.407254000
C	0.000000000	-6.163398000	0.726241000
C	0.000000000	-6.163398000	-0.726241000
C	0.000000000	-4.921492000	-1.407254000
H	0.000000000	-4.922651000	-2.494725000
H	0.000000000	-4.922651000	2.494725000
C	0.000000000	-7.391373000	1.405158000
C	0.000000000	-8.614196000	0.723594000
C	0.000000000	-8.614196000	-0.723594000
H	0.000000000	-7.391896000	2.492744000
C	0.000000000	-7.391373000	-1.405158000
H	0.000000000	-7.391896000	-2.492744000
C	0.000000000	-9.869210000	1.407121000
C	0.000000000	-11.050702000	0.712824000
H	0.000000000	-11.996631000	1.246190000
C	0.000000000	-11.050702000	-0.712824000
H	0.000000000	-11.996631000	-1.246190000
C	0.000000000	-9.869210000	-1.407121000
H	0.000000000	-9.867126000	-2.493975000
H	0.000000000	-9.867126000	2.493975000

Decacene

C	0.000000000	8.621906000	1.405956000
C	0.000000000	9.841124000	0.724750000
C	0.000000000	9.841124000	-0.724750000
C	0.000000000	8.621906000	-1.405956000
C	0.000000000	7.390389000	-0.726790000
C	0.000000000	7.390389000	0.726790000
H	0.000000000	8.622264000	2.493528000
H	0.000000000	8.622264000	-2.493528000
C	0.000000000	6.152705000	1.407566000
C	0.000000000	4.934750000	0.729827000
C	0.000000000	4.934750000	-0.729827000
C	0.000000000	6.152705000	-1.407566000
H	0.000000000	6.153481000	-2.495027000
H	0.000000000	6.153481000	2.495027000
C	0.000000000	11.098497000	1.408089000
C	0.000000000	12.278212000	0.713906000
H	0.000000000	13.224600000	1.246463000
C	0.000000000	12.278212000	-0.713906000
H	0.000000000	13.224600000	-1.246463000
C	0.000000000	11.098497000	-1.408089000
H	0.000000000	11.096485000	-2.494933000
H	0.000000000	11.096485000	2.494933000
C	0.000000000	3.687735000	1.409174000
C	0.000000000	2.471292000	0.733532000

C	0.000000000	2.471292000	-0.733532000
C	0.000000000	3.687735000	-1.409174000
H	0.000000000	3.688822000	-2.496620000
H	0.000000000	3.688822000	2.496620000
C	0.000000000	1.228172000	1.410629000
C	0.000000000	0.000000000	0.735589000
C	0.000000000	0.000000000	-0.735589000
C	0.000000000	1.228172000	-1.410629000
H	0.000000000	1.228830000	-2.498051000
H	0.000000000	1.228830000	2.498051000
C	0.000000000	-1.228172000	1.410629000
C	0.000000000	-2.471292000	0.733532000
C	0.000000000	-2.471292000	-0.733532000
C	0.000000000	-1.228172000	-1.410629000
H	0.000000000	-1.228830000	-2.498051000
H	0.000000000	-1.228830000	2.498051000
C	0.000000000	-3.687735000	1.409174000
C	0.000000000	-4.934750000	0.729827000
C	0.000000000	-4.934750000	-0.729827000
C	0.000000000	-3.687735000	-1.409174000
H	0.000000000	-3.688822000	-2.496620000
H	0.000000000	-3.688822000	2.496620000
C	0.000000000	-6.152705000	1.407566000
C	0.000000000	-7.390389000	0.726790000
C	0.000000000	-7.390389000	-0.726790000
H	0.000000000	-6.153481000	2.495027000
C	0.000000000	-6.152705000	-1.407566000
H	0.000000000	-6.153481000	-2.495027000
C	0.000000000	-8.621906000	1.405956000
C	0.000000000	-9.841124000	0.724750000
C	0.000000000	-9.841124000	-0.724750000
C	0.000000000	-8.621906000	-1.405956000
H	0.000000000	-8.622264000	-2.493528000
H	0.000000000	-8.622264000	2.493528000
C	0.000000000	-11.098497000	1.408089000
C	0.000000000	-12.278212000	0.713906000
H	0.000000000	-13.224600000	1.246463000
C	0.000000000	-12.278212000	-0.713906000
H	0.000000000	-13.224600000	-1.246463000
C	0.000000000	-11.098497000	-1.408089000
H	0.000000000	-11.096485000	-2.494933000
H	0.000000000	-11.096485000	2.494933000

Undecacene

C	0.000000000	9.852169000	1.406651000
C	0.000000000	11.068749000	0.725669000
C	0.000000000	11.068749000	-0.725669000
C	0.000000000	9.852169000	-1.406651000
C	0.000000000	8.617778000	-0.727441000
C	0.000000000	8.617778000	0.727441000
H	0.000000000	9.852720000	2.494205000
H	0.000000000	9.852720000	-2.494205000
C	0.000000000	7.383727000	1.407983000
C	0.000000000	6.163203000	0.729711000
C	0.000000000	6.163203000	-0.729711000
C	0.000000000	7.383727000	-1.407983000
H	0.000000000	7.384491000	-2.495432000
H	0.000000000	7.384491000	2.495432000
C	0.000000000	12.327811000	1.408800000
C	0.000000000	13.506314000	0.714656000
H	0.000000000	14.452930000	1.246814000
C	0.000000000	13.506314000	-0.714656000
H	0.000000000	14.452930000	-1.246814000
C	0.000000000	12.327811000	-1.408800000
H	0.000000000	12.326093000	-2.495630000
H	0.000000000	12.326093000	2.495630000
C	0.000000000	4.918446000	1.409116000

C	0.000000000	3.703080000	0.732571000
C	0.000000000	3.703080000	-0.732571000
C	0.000000000	4.918446000	-1.409116000
H	0.000000000	4.919359000	-2.496565000
H	0.000000000	4.919359000	2.496565000
C	0.000000000	2.457098000	1.410363000
C	0.000000000	1.235674000	0.735315000
C	0.000000000	1.235674000	-0.735315000
C	0.000000000	2.457098000	-1.410363000
H	0.000000000	2.458000000	-2.497806000
H	0.000000000	2.458000000	2.497806000
C	0.000000000	0.000000000	1.411029000
C	0.000000000	-1.235674000	0.735315000
C	0.000000000	-1.235674000	-0.735315000
C	0.000000000	0.000000000	-1.411029000
H	0.000000000	0.000000000	-2.498460000
H	0.000000000	0.000000000	2.498460000
C	0.000000000	-2.457098000	1.410363000
C	0.000000000	-3.703080000	0.732571000
C	0.000000000	-3.703080000	-0.732571000
C	0.000000000	-2.457098000	-1.410363000
H	0.000000000	-2.458000000	-2.497806000
H	0.000000000	-2.458000000	2.497806000
C	0.000000000	-4.918446000	1.409116000
C	0.000000000	-6.163203000	0.729711000
C	0.000000000	-6.163203000	-0.729711000
H	0.000000000	-4.919359000	2.496565000
C	0.000000000	-4.918446000	-1.409116000
H	0.000000000	-4.919359000	-2.496565000
C	0.000000000	-7.383727000	1.407983000
C	0.000000000	-8.617778000	0.727441000
C	0.000000000	-8.617778000	-0.727441000
C	0.000000000	-7.383727000	-1.407983000
H	0.000000000	-7.384491000	-2.495432000
H	0.000000000	-7.384491000	2.495432000
C	0.000000000	-9.852169000	1.406651000
C	0.000000000	-11.068749000	0.725669000
C	0.000000000	-11.068749000	-0.725669000
C	0.000000000	-9.852169000	-1.406651000
H	0.000000000	-9.852720000	-2.494205000
H	0.000000000	-9.852720000	2.494205000
C	0.000000000	-12.327811000	1.408800000
C	0.000000000	-13.506314000	0.714656000
H	0.000000000	-14.452930000	1.246814000
C	0.000000000	-13.506314000	-0.714656000
H	0.000000000	-14.452930000	-1.246814000
C	0.000000000	-12.327811000	-1.408800000
H	0.000000000	-12.326093000	-2.495630000
H	0.000000000	-12.326093000	2.495630000

Dodecacene

C	0.000000000	11.082077000	1.407258000
C	0.000000000	12.296456000	0.726447000
C	0.000000000	12.296456000	-0.726447000
C	0.000000000	11.082077000	-1.407258000
C	0.000000000	9.845188000	-0.728050000
C	0.000000000	9.845188000	0.728050000
H	0.000000000	11.082579000	2.494801000
H	0.000000000	11.082579000	-2.494801000
C	0.000000000	8.614367000	1.408456000
C	0.000000000	7.391119000	0.729818000
C	0.000000000	7.391119000	-0.729818000
C	0.000000000	8.614367000	-1.408456000
H	0.000000000	8.614986000	-2.495886000
H	0.000000000	8.614986000	2.495886000
C	0.000000000	13.556939000	1.409411000
C	0.000000000	14.734414000	0.715311000

H	0.000000000	15.681312000	1.246965000
C	0.000000000	14.734414000	-0.715311000
H	0.000000000	15.681312000	-1.246965000
C	0.000000000	13.556939000	-1.409411000
H	0.000000000	13.555266000	-2.496235000
H	0.000000000	13.555266000	2.496235000
C	0.000000000	6.149233000	1.409269000
C	0.000000000	4.933173000	0.731905000
C	0.000000000	4.933173000	-0.731905000
C	0.000000000	6.149233000	-1.409269000
H	0.000000000	6.149899000	-2.496703000
H	0.000000000	6.149899000	2.496703000
C	0.000000000	3.686761000	1.410179000
C	0.000000000	2.469394000	0.734490000
C	0.000000000	2.469394000	-0.734490000
C	0.000000000	3.686761000	-1.410179000
H	0.000000000	3.687594000	-2.497622000
H	0.000000000	3.687594000	2.497622000
C	0.000000000	1.228173000	1.411034000
C	0.000000000	0.000000000	0.735884000
C	0.000000000	0.000000000	-0.735884000
C	0.000000000	1.228173000	-1.411034000
H	0.000000000	1.228637000	-2.498471000
H	0.000000000	1.228637000	2.498471000
C	0.000000000	-1.228173000	1.411034000
C	0.000000000	-2.469394000	0.734490000
C	0.000000000	-2.469394000	-0.734490000
C	0.000000000	-1.228173000	-1.411034000
H	0.000000000	-1.228637000	-2.498471000
H	0.000000000	-1.228637000	2.498471000
C	0.000000000	-3.686761000	1.410179000
C	0.000000000	-4.933173000	0.731905000
C	0.000000000	-4.933173000	-0.731905000
H	0.000000000	-3.687594000	2.497622000
C	0.000000000	-3.686761000	-1.410179000
H	0.000000000	-3.687594000	-2.497622000
C	0.000000000	-6.149233000	1.409269000
C	0.000000000	-7.391119000	0.729818000
C	0.000000000	-7.391119000	-0.729818000
C	0.000000000	-6.149233000	-1.409269000
H	0.000000000	-6.149899000	-2.496703000
H	0.000000000	-6.149899000	2.496703000
C	0.000000000	-8.614367000	1.408456000
C	0.000000000	-9.845188000	0.728050000
C	0.000000000	-9.845188000	-0.728050000
C	0.000000000	-8.614367000	-1.408456000
H	0.000000000	-8.614986000	-2.495886000
H	0.000000000	-8.614986000	2.495886000
C	0.000000000	-11.082077000	1.407258000
C	0.000000000	-12.296456000	0.726447000
C	0.000000000	-12.296456000	-0.726447000
C	0.000000000	-11.082077000	-1.407258000
H	0.000000000	-11.082579000	-2.494801000
H	0.000000000	-11.082579000	2.494801000
C	0.000000000	-13.556939000	1.409411000
C	0.000000000	-14.734414000	0.715311000
H	0.000000000	-15.681312000	1.246965000
C	0.000000000	-14.734414000	-0.715311000
H	0.000000000	-15.681312000	-1.246965000
C	0.000000000	-13.556939000	-1.409411000
H	0.000000000	-13.555266000	-2.496235000
H	0.000000000	-13.555266000	2.496235000

Absolute energies in hartrees

For CAS(2,2)

CASSCF	Singlet	triplet(V)	triplet(ad)
Napthalene	-383.38441	-383.24665	-383.26936
Anthracene	-536.04293	-535.94541	-535.96291
Tetracene	-688.68155	-688.60746	-688.62182
Pentacene	-841.33302	-841.27290	-841.28592
Hexacene	-993.96786	-993.92754	-993.93686
Heptacene	-1146.61337	-1146.58385	-1146.58902
Octacene	-1299.24731	-1299.23143	-1299.23507
Nonacene	-1451.89188	-1451.87789	-1451.88164
Decacene	-1604.52800	-1604.52054	-1604.52478
Undecacene	-1757.17044	-1757.16272	-1757.16831
Dodecacene	-1909.80722	-1909.80271	-1909.80970

CASPT2	Singlet	triplet(V)	triplet(ad)
Napthalene	-384.68168	-384.55890	-384.57305
Anthracene	-537.85035	-537.75451	-537.76765
Tetracene	-691.01508	-690.95007	-690.96023
Pentacene	-844.17951	-844.12497	-844.13404
Hexacene	-997.34530	-997.30762	-997.31364
Heptacene	-1150.50872	-1150.47927	-1150.48107
Octacene	-1303.67513	-1303.65287	-1303.65343
Nonacene	-1456.83681	-1456.81874	-1456.81848
Decacene	-1610.00377	-1609.98748	-1609.98718
Undecacene	-1763.16512	-1763.15176	-1763.15100
Dodecacene	-1916.33239	-1916.31851	-1916.31795

tPBE	Singlet	triplet(V)	triplet(ad)
Napthalene	-385.34215	-385.22345	-385.23633
Anthracene	-538.76306	-538.66810	-538.68056
Tetracene	-692.18702	-692.12778	-692.13636
Pentacene	-845.60431	-845.55449	-845.56218
Hexacene	-999.02844	-998.99703	-999.00166
Heptacene	-1152.44466	-1152.42211	-1152.42291
Octacene	-1305.86949	-1305.85412	-1305.85395
Nonacene	-1459.28399	-1459.27467	-1459.27355
Decacene	-1612.71001	-1612.70148	-1612.70036
Undecacene	-1766.12452	-1766.12129	-1766.11934
Dodecacene	-1919.55137	-1919.54583	-1919.54403

For CAS(4,4)

CASSCF	Singlet	triplet(V)	triplet(ad)
Napthalene	-383.40398	-383.25463	-383.27593
Anthracene	-536.06138	-535.95035	-535.96659
Tetracene	-688.69766	-688.61400	-688.62460
Pentacene	-841.33615	-841.28384	-841.29545
Hexacene	-993.99398	-993.94730	-993.95675
Heptacene	-1146.61616	-1146.59821	-1146.60234
Octacene	-1299.27621	-1299.25022	-1299.25386
Nonacene	-1451.91440	-1451.89430	-1451.89699
Decacene	-1604.55768	-1604.53876	-1604.54295
Undecacene	-1757.19593	-1757.18062	-1757.18486
Dodecacene	-1909.83285	-1909.81978	-1909.82750

CASPT2	Singlet	triplet(V)	triplet(ad)
Napthalene	-384.68369	-384.56340	-384.57542
Anthracene	-537.85003	-537.75387	-537.76726
Tetracene	-691.01554	-690.94971	-690.96000
Pentacene	-844.18331	-844.13457	-844.14274
Hexacene	-997.34697	-997.30518	-997.31144
Heptacene	-1150.51266	-1150.48607	-1150.48820
Octacene	-1303.67479	-1303.65029	-1303.65097
Nonacene	-1456.84012	-1456.82426	-1456.82444
Decacene	-1610.00163	-1609.98492	-1609.98465
Undecacene	-1763.16848	-1763.15659	-1763.15633
Dodecacene	-1916.33276	-1916.32575	-1916.31547

tPBE	Singlet	triplet(V)	triplet(ad)
Napthalene	-385.33662	-385.22400	-385.23686
Anthracene	-538.75703	-538.66788	-538.68085
Tetracene	-692.18109	-692.12711	-692.13629
Pentacene	-845.60450	-845.56379	-845.57073
Hexacene	-999.02092	-998.98556	-998.99079
Heptacene	-1152.44491	-1152.42599	-1152.42760
Octacene	-1305.85877	-1305.84249	-1305.84265
Nonacene	-1459.28019	-1459.27560	-1459.27549
Decacene	-1612.69711	-1612.68970	-1612.68885
Undecacene	-1766.12169	-1766.12039	-1766.11968
Dodecacene	-1919.54027	-1919.54869	-1919.53245

For CAS(8,8)

CASSCF	Singlet	triplet(V)	triplet(ad)
Napthalene	-383.46659	-383.34608	-383.35950
Anthracene	-536.09568	-535.99774	-536.01438
Tetracene	-688.73530	-688.67326	-688.68986
Pentacene	-841.36133	-841.29743	-841.30748
Hexacene	-994.02101	-993.99383	-994.00486
Heptacene	-1146.66233	-1146.65091	-1146.65600
Octacene	-1299.30846	-1299.28774	-1299.29031
Nonacene	-1451.96598	-1451.94740	-1451.95059
Decacene	-1604.58741	-1604.57171	-1604.57536
Undecacene	-1757.23577	-1757.21385	-1757.21927
Dodecacene	-1909.87378	-1909.85621	-1909.86316

CASPT2	Singlet	triplet(V)	triplet(ad)
Napthalene	-384.68084	-384.56245	-384.57602
Anthracene	-537.85173	-537.76562	-537.77641
Tetracene	-691.01461	-690.94541	-690.95525
Pentacene	-844.18419	-844.13889	-844.14776
Hexacene	-997.34710	-997.30177	-997.30781
Heptacene	-1150.51076	-1150.48199	-1150.48451
Octacene	-1303.67599	-1303.65812	-1303.65920
Nonacene	-1456.83516	-1456.81960	-1456.82008
Decacene	-1610.00590	-1609.98738	-1609.98710
Undecacene	-1763.17167	-1763.15949	-1763.16033
Dodecacene	-1916.33501	-1916.32964	-1916.31788

tPBE	Singlet	triplet(V)	triplet(ad)
Napthalene	-385.32260	-385.20142	-385.21720
Anthracene	-538.75186	-538.66910	-538.67862
Tetracene	-692.17368	-692.10599	-692.11416
Pentacene	-845.59976	-845.56211	-845.57057
Hexacene	-999.01628	-998.97418	-998.97869
Heptacene	-1152.43525	-1152.40599	-1152.40761
Octacene	-1305.85420	-1305.84204	-1305.84265
Nonacene	-1459.26513	-1459.25431	-1459.25435
Decacene	-1612.69375	-1612.68282	-1612.68223
Undecacene	-1766.11500	-1766.11364	-1766.11330
Dodecacene	-1919.53320	-1919.54177	-1919.52469

For FP-1

GASSCF	Singlet	triplet(V)	triplet(ad)
Napthalene	-383.42852	-383.29303	-383.31940
Anthracene	-536.16416	-536.05822	-536.07627
Tetracene	-688.83438	-688.76070	-688.77688
Pentacene	-841.52058	-841.45185	-841.46669
Hexacene	-994.18405	-994.14006	-994.15141
Heptacene	-1146.85961	-1146.82431	-1146.83045
Octacene	-1299.52062	-1299.50173	-1299.50612
Nonacene	-1452.19170	-1452.17439	-1452.17850
Decacene	-1604.85446	-1604.84384	-1604.84851
Undecacene	-1757.52079	-1757.51051	-1757.51645
Dodecacene	-1910.18297	-1910.17512	-1910.18267

AGASPT2	Singlet	triplet(V)	triplet(ad)
Napthalene	-384.64265	-384.51386	-384.53539
Anthracene	-537.82765	-537.73169	-537.74447
Tetracene	-690.98007	-690.90967	-690.92185
Pentacene	-844.14176	-844.08079	-844.09170

MC-PDFT	Singlet	triplet(V)	triplet(ad)
Napthalene	-385.32883	-385.20518	-385.21634
Anthracene	-538.74599	-538.66423	-538.67341
Tetracene	-692.16847	-692.10632	-692.11488
Pentacene	-845.59172	-845.54438	-845.55138
Hexacene	-999.01503	-998.97854	-998.98356
Heptacene	-1152.43957	-1152.41201	-1152.41330
Octacene	-1305.86143	-1305.84163	-1305.84169
Nonacene	-1459.28711	-1459.26891	-1459.26831
Decacene	-1612.70812	-1612.69586	-1612.69498
Undecacene	-1766.13513	-1766.12130	-1766.12008
Dodecacene	-1919.55678	-1919.54735	-1919.54601

For DFP-1

GASSCF	Singlet	triplet(V)	triplet(ad)
Napthalene	-383.46536	-383.35270	-383.36636
Anthracene	-536.15790	-536.04772	-536.06574
Tetracene	-688.81218	-688.74444	-688.75988
Pentacene	-841.49863	-841.43850	-841.45152
Hexacene	-994.16986	-994.13407	-994.14494
Heptacene	-1146.84574	-1146.81211	-1146.81764
Octacene	-1299.52880	-1299.49801	-1299.50221
Nonacene	-1452.18885	-1452.16498	-1452.16852
Decacene	-1604.86611	-1604.84218	-1604.84668
Undecacene	-1757.52475	-1757.50377	-1757.50895
Dodecacene	-1910.19664	-1910.17509	-1910.18249

tPBE	Singlet	triplet(V)	triplet(ad)
Napthalene	-385.31312	-385.19052	-385.21335
Anthracene	-538.73815	-538.66151	-538.67107
Tetracene	-692.16532	-692.10665	-692.11292
Pentacene	-845.58346	-845.53586	-845.54382
Hexacene	-999.00880	-998.96359	-998.96922
Heptacene	-1152.42549	-1152.40314	-1152.40483
Octacene	-1305.84635	-1305.82608	-1305.82654
Nonacene	-1459.26961	-1459.25954	-1459.25954
Decacene	-1612.69039	-1612.67987	-1612.67945
Undecacene	-1766.11727	-1766.11159	-1766.11130
Dodecacene	-1919.53805	-1919.53108	-1919.53031

AGASPT2	Singlet	triplet(V)	triplet(ad)
Napthalene	-384.65655	-384.53710	-384.55141
Anthracene	-537.81720	-537.72177	-537.73452
Tetracene	-690.96388	-690.89766	-690.90578

For WFT-1

GASSCF	Singlet	triplet(V)	triplet(ad)
Napthalene	-383.46994	-383.35492	-383.36801
Anthracene	-536.16247	-536.05122	-536.06834
Tetracene	-688.81351	-688.74654	-688.76264
Pentacene	-841.51670	-841.44495	-841.45741
Hexacene	-994.18889	-994.13882	-994.14939
Heptacene	-1146.85741	-1146.81828	-1146.82354
Octacene	-1299.53553	-1299.50290	-1299.50686
Nonacene	-1452.19433	-1452.17114	-1452.17442
Decacene	-1604.87084	-1604.84710	-1604.85132
Undecacene	-1757.52981	-1757.51001	-1757.51478
Dodecacene	-1910.20070	-1910.17998	-1910.18703

tPBE	Singlet	triplet(V)	triplet(ad)
Napthalene	-385.31436	-385.19100	-385.21389
Anthracene	-538.73953	-538.66319	-538.67225
Tetracene	-692.16565	-692.10851	-692.11363
Pentacene	-845.58234	-845.53712	-845.54519
Hexacene	-999.00603	-998.96733	-998.97243
Heptacene	-1152.42854	-1152.40485	-1152.40648
Octacene	-1305.85116	-1305.83031	-1305.83033
Nonacene	-1459.27190	-1459.26180	-1459.26156
Decacene	-1612.69512	-1612.68444	-1612.68349
Undecacene	-1766.11950	-1766.11445	-1766.11362
Dodecacene	-1919.54218	-1919.53586	-1919.53443

AGASPT2	Singlet	triplet(V)	triplet(ad)
Napthalene	-384.66015	-384.53869	-384.55278
Anthracene	-537.82078	-537.72510	-537.73693
Tetracene	-690.96526	-690.90002	-690.90805

For WFP-3

GASSCF	Singlet	triplet(V)	triplet(ad)
Napthalene	-383.49481	-383.37872	-383.39382
Anthracene	-536.18471	-536.09245	-536.10657
Tetracene	-688.86592	-688.79335	-688.80562
Pentacene	-841.54504	-841.49092	-841.50183
Hexacene	-994.22011	-994.17935	-994.18764
Heptacene	-1146.89068	-1146.86368	-1146.86752
Octacene	-1299.56060	-1299.54082	-1299.54349
Nonacene	-1452.22993	-1452.21426	-1452.21656
Decacene	-1604.89680	-1604.88381	-1604.88643
Undecacene	-1757.56376	-1757.55139	-1757.55492

AGASPT2	Singlet	triplet(V)	triplet(ad)
Napthalene	-384.67667	-384.55800	-384.57190
Anthracene	-537.83968	-537.75349	-537.76549
Tetracene	-690.99908	-690.93465	-690.94507

tPBE	Singlet	triplet(V)	triplet(ad)
Napthalene	-385.31944	-385.20012	-385.21628
Anthracene	-538.74220	-538.66183	-538.67347
Tetracene	-692.16680	-692.11146	-692.12083
Pentacene	-845.59017	-845.55034	-845.55743
Hexacene	-999.01368	-998.98612	-998.98973
Heptacene	-1152.43896	-1152.42209	-1152.42295
Octacene	-1305.86155	-1305.85300	-1305.85142
Nonacene	-1459.28822	-1459.28124	-1459.28023
Decacene	-1612.71628	-1612.71044	-1612.70815
Undecacene	-1766.13839	-1766.13582	-1766.13351

[1] B. Hajgat, M. Huzak, and M. S. Deleuze, *J. Phys. Chem. A*, 2011, **115**, 9282–9293.
[2] J. Fosso-Tande, T. Nguyen, G. Gidofalvi, and A. E. DePrince III, *J. Chem. Theory Comput.*, 2016, **12,** 2260-2271.
